# Role of RGS17 in cisplatin-induced cochlear inflammation and ototoxicity via caspase-3 activation

**DOI:** 10.3389/fimmu.2025.1470625

**Published:** 2025-02-21

**Authors:** Raheem F. H. Al Aameri, Entkhab M. A. Alanisi, Dheyaa Al Sallami, Ian Alberts, Shelley Tischkau, Leonard P. Rybak, Vickram Ramkumar

**Affiliations:** ^1^ Department of Pharmacology, Southern Illinois University School of Medicine, Springfield, IL, United States; ^2^ Department of Biology, Wasit University, College of Applied Science, Wasit, Iraq; ^3^ Department of Biology, Mustansiriyah University, College of Science, Baghdad, Iraq; ^4^ Southern Illinois University School of Medicine, Springfield, IL, United States

**Keywords:** cisplatin, *RGS17*, *CXCL1*, hair cells, inflammation

## Abstract

Cisplatin is a chemotherapy drug used to treat different solid tumors, including ovarian, bladder, lung, and head and neck cancers. One of its significant side effects is ototoxicity, especially when high doses are required. Cisplatin-induced ototoxicity is associated with increased cochlear cell death resulting from DNA damage, caspase activation, oxidative stress, inflammation, and glutamate excitotoxicity. The regulator of G protein signaling 17 (RGS17), a member of the RGS-RZ subfamily, hastens the hydrolysis of GTP to GDP on the G_α_ subunit. In the current study, we demonstrate the role of *RGS17* in cisplatin-induced cochlear inflammation and ototoxicity. C57BL/6J mice treated with two cycles of cisplatin (3.5 mg/kg) showed a significant elevation in ABR thresholds, along with loss of outer hair cells and inner hair cells synapse. Furthermore, immunohistochemical analysis revealed that cisplatin administration upregulates CXCL1, accompanied by an increase in the number of CD45 and CD68-positive immune cells. On the other hand, *RGS17* knockout in hair cells protects against cisplatin-induced elevation of ABR thresholds, outer hair cell loss, cochlear inflammation, and inner hair cell synaptopathy. Moreover, *RGS17* knockout downregulates CXCL1 immunolabeling and decreases the number of CD45 and CD68-positive immune cells induced by cisplatin. These results suggest that *RGS17* is implicated in cisplatin ototoxicity, potentially by initiating the immune cascade, and indicate *RGS17* as a relevant target for treating cisplatin ototoxicity.

## Introduction

Cisplatin is a chemotherapeutic agent used to treat different cancers like head and neck, lung, bladder, and reproductive system (1). However, cisplatin use is associated with unfavorable side effects including neurotoxicity (2), nephrotoxicity (3), and ototoxicity (4). More than 60% of pediatric patients receiving cisplatin report renal dysfunction, and over 60% experience permanent bilateral sensorineural hearing loss (5). Cisplatin-induced hearing loss is usually bilateral and irreversible (6). The ototoxic effects associated with cisplatin administration include DNA damage, reactive oxygen species (ROS) production, activation of cytoplasmic caspases, and mitochondrial dysfunction of cochlear cells (7–9). Cisplatin affects various cell types in the cochlea including outer hair cells (OHCs) in the organ of Corti (OC), stria vascularis (SV), spiral ligament (SL), and spiral ganglion neuron (SGN) cells (10). Treating ototoxicity remains a significant challenge, not only due to the incomplete understanding of the mechanisms behind hearing loss but also because of the difficulties in effectively delivering protective agents to the inner ear (11, 12).

Cochlear inflammation has been linked with hearing loss (13). Following trauma, tumor necrosis factor-α (TNF-α), interleukin 6 (IL-6), chemokine (C-X-C motif) ligand 1 (CXCL1), macrophage inflammatory peptide 2 (MIP-2), soluble intercellular adhesion molecule-1 (sICAM-1), and vascular endothelial growth factor (VEGF) are increased in the cochlea (14, 15). These mediators recruit immune cells to the site of trauma and tissue injury (16). Activation of CXCL1 in the cochlea disturbs cochlear function as evidenced by altered auditory brainstem-evoked potential and reductions in wave I supra-threshold amplitudes. This is accompanied by the migration of CD45 and CD68-positive immune cells into the cochlea. However, inhibition of CXCR2 (CXCL1 receptor) ameliorates cisplatin-induced ototoxicity (4).

As membrane-bound receptors, G protein-coupled receptors (GPCRs) like cannabinoid receptor 2 (CB2R) exert otoprotective activity. GPCRs transfer the signals from the external environment to the inside of the cell. Agonist binding to GPCRs drives the activation of intracellular first messengers (effectors) and subsequent second messenger activation (protein kinase) to influence cellular functions (17). Previously, our lab has shown that GPCRs such as CB2R and A1 adenosine receptor (A1AR) are not only expressed in the cochlea but also are protective against cisplatin-induced OHC loss, inner hair cell synaptopathy, and ABR threshold shifts, through decreased inflammation and reduced apoptosis in the cochlear cells (4, 18–21). GPCRs regulate the activity of heterotrimeric G proteins (22). Interestingly, G proteins like G_i_, G_o_, G_s_, G_q_, and G_z_ are expressed throughout the cochlea, highlighting their important role in GPCR-mediated signaling within auditory sensory structures (23–26). Inactive G proteins are bound to GDP, and their activation requires the conversion of GDP to GTP, which occurs upon GPCR stimulation to modulate downstream signaling (27, 28). Regulators of G protein signaling (RGS) are a multifunctional and highly diverse group of proteins that negatively regulate GPCR signaling (29). RGS act as GTPase accelerating proteins (GAP) which hasten the hydrolysis of active GTP-bound G proteins, promote the formation of GDP, and terminate the action of the associated GPCR. RGS17 is a member of the RGS-RZ subfamily, which targets GTP-bound Gα_i1–3_, G_αo_, G_αz_, and G_αq_ for hydrolysis (30). Studies have shown that RGS17 was upregulated in lung and prostate cancers (31–33), as well as in the cochlea following cisplatin treatment (19). This highlights *RGS17* and other RGS genes as potential targets for treating cisplatin toxicity. Cochlear RNA sequencing from our laboratory verifies differential expression of the RGS genes, including *RGS17*, after cisplatin injection in rats (18). The current study implicates RGS17 as a key player in cisplatin-induced ototoxicity, as it promotes cochlear inflammation in response to cisplatin. Accordingly, knockout of *RGS17* ameliorates proinflammatory pathways, notably CXCL1, induced by cisplatin.

## Materials and methods

### Generation of *RGS17* tissue-specific knockout mice

**Figure 1:**
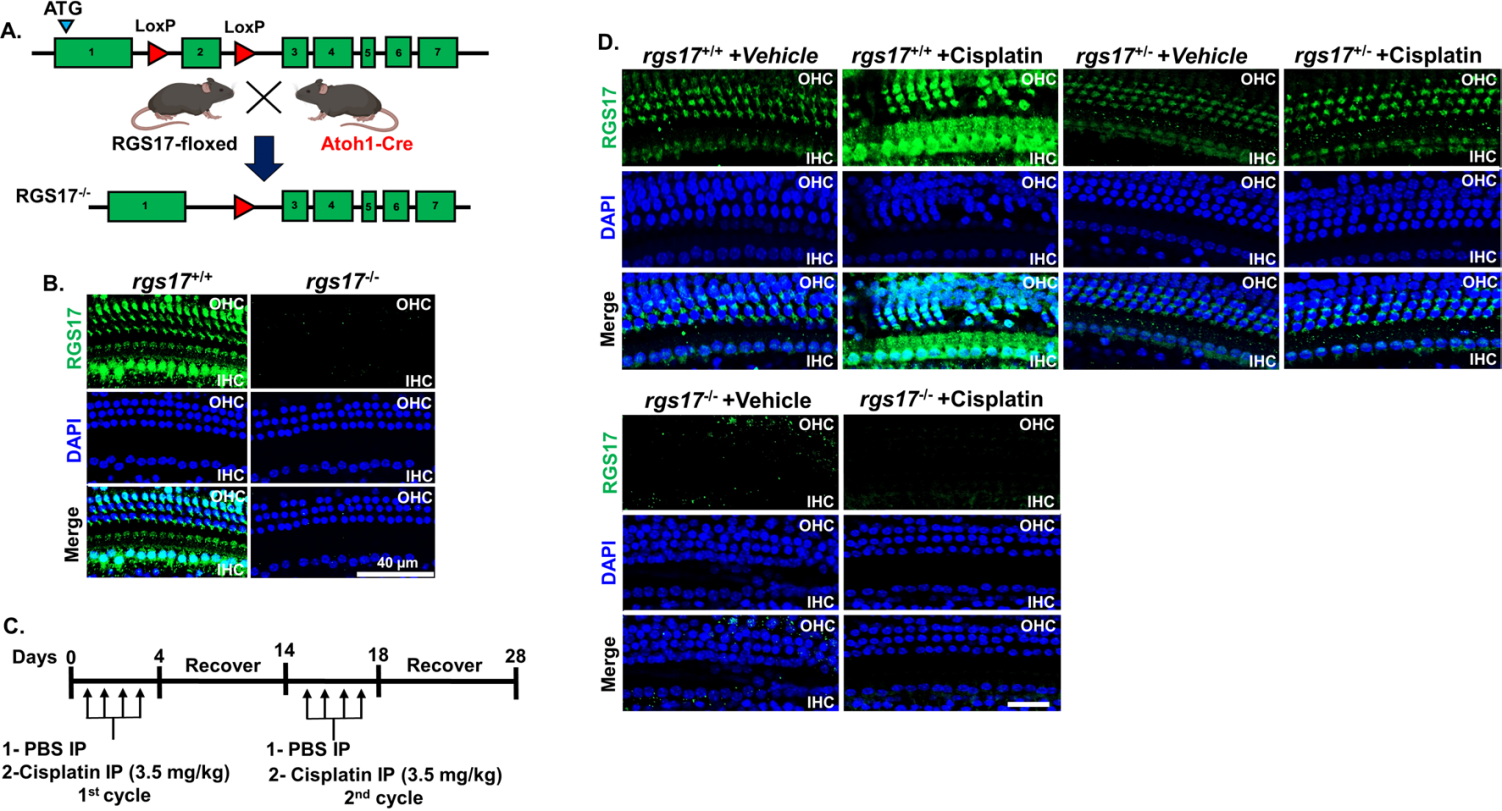
RGS17 tissue-specific knockout scheme and effect of cisplatin in RGS17 expression in whole-mount dissection. **(A)** Genomic organization of *RGS17^loxp^* and *RGS17^−/−^* alleles. Exon 2 was flanked with the *loxP* sequence, which was cleaved using Atoh-1CreEr to generate mice with specific RGS17^−/−^ knockout in the hair cells. **(B)** The cochleae were harvested from wild-type RGS17^+/+^ and RGS17^−/−^ mice. Whole-mount dissection for basal turn was immunolabeled with RGS17 antibody to validate RGS17 expression. The expression of RGS17 (green) was located in the outer and inner hair cells of wild-type mice. However, RGS17 was not detected in the outer and inner hair cells of knockout mice. **(C)** Schematic illustration of the experimental design that describes the dosage and route of administration of control or cisplatin (3.5 mg/kg) for two cycles; each cycle consists of a 4-day cisplatin administration followed by a 10-day recovery period. Mice were sacrificed at the end of the second cycle **(D)**, and cochleae were collected and processed for whole-mount dissection. The basal turns of each cochlea were immunolabeled with RGS17 (green) and DAPI (blue). High levels of RGS17 immunolabeling were detected in the outer and inner hair cells of wild-type RGS17^+/+^ mice treated with cisplatin. Inducible hair cell-specific RGS17 knockdown (RGS17^+/−^) mice showed a slight increase of RGS17 immunolabeling in the outer and inner hair cells following cisplatin administration. However, a hair cell-specific knockout of RGS17 abolished the immunolabeling of RGS17 in both the outer and inner hair cells. Figures shown are representative of six independent animals per treatment group. Scale bar = 40 µm.

RGS17 mice with *LoxP* site gene modifications were crossed with tamoxifen-induced *Atoh1-CreER* mice from Brandon Cox, Ph.D. (SIU School of Medicine), to generate inducible hair cell-specific RGS17 knockout. We have generated *C57BL/6J-RGS17^loxP/loxP^* mouse by flanking *RGS17* exon 2 in the hair cells with *LoxP* sites, Jackson Laboratory (Bar Harbor, ME). No such line is available commercially or from private sources. Heterozygous *C57BL/6J-RGS17^loxP/+^* mice were mated to generate homozygous *C57BL/6J-RGS17^loxP/loxP^* mice which were subsequently interbred with *Atoh1-CreER* to generate *C57BL/6J:Atoh1-CreER: RGS17^loxP/+^*. These *C57BL/6J:Atoh1-CreER: RGS17^loxP/+^* mice were then mated with *RGS17^loxP/loxP^* or *RGS17^loxP/+^* to generate *C57BL/6J:Atoh1-CreER: RGS17^loxP/+^* or *C57BL/6J:Atoh1-CreER: RGS17^loxP/loxP^* (**Figure 1A**). Both male and female mice were used in all studies. Animal protocols were approved by the Southern Illinois University School of Medicine, Institutional Animal Care and Use Committee (IACUC).

### Tamoxifen treatment

To knockout RGS17 in the hair cells, *C57BL/6J:Atoh1-CreER: RGS17^loxP/loxP^* and *C57BL/6J:Atoh1-CreER: RGS17^loxP/+^* mice were injected with tamoxifen [3 mg/40 g intraperitoneally (IP)] at P0 and P1 (20–24 h apart). Control mice did not receive tamoxifen injection and were kept separately from the tamoxifen-treated mice.

### Genotyping

Tail snip was collected from mice at P10 and used to genotype mice by TransnetYX company (Cordova, TN). In brief, DNA was extracted from the tail snip, and *RGS17* null was determined using the following primers: sense 5′-*CGTATAGCATACATTATACGAAGTTA TGTTGCGTT*-3′, antisense 5′-*CAGAAAATATTAAGTCATGAAGAGCCTGG*-3′, and probe 5′-*AAGAGAAACACGGTTAGACA*-3′. *Atoh1-CreER* was identified using the following primers: sense 5′-*TTAATCCATATTGGCAGAACGAAAACG*-3′, antisense 5′-*CAGGCTAAGTGCCTTCTCTACA*-3, and probe 5′-*CCTGCGGTGCTAACC*-3.

### Animal procedure and cisplatin treatment

Mice were housed in accordance with the standards established by the Division of Laboratory Animal Medicine (DLAM) facility of SIU School of Medicine. They were kept in a controlled environment with a 12:12-h light:dark cycle, with *ad libitum* access to food and water. Each group consisted of eight animals. Mice were anesthetized using a mixture of ketamine (90 g/kg) and xylazine (17mg/kg), administered intraperitoneally, and the depth of anesthesia was confirmed by the absence of reflex to the toe pinch. Auditory brainstem responses (ABRs) were examined in a soundproof chamber. Cisplatin (3.5 mg/kg) or equivalent volumes of vehicle were injected for two cycles, each cycle consisted of a 4-day injection followed by a 10-day recovery period (the cumulative dose is 28 mg/kg). Pre-ABRs were recorded on day 0, while post-ABRs were recorded on days 14 and 28. The mice used in our experiments were 6 weeks old at the time of study started.

### Auditory brainstem responses

High-Frequency Intelligent Hearing Systems (HIS) was used to conduct ABRs in mice as described previously (34). Mice were anesthetized using a ketamine/xylazine mixture and placed in a soundproof chamber, with electrodes placed as follows: negative electrodes were inserted under the pinna of each ear, a ground electrode was inserted in the hind flank muscle, and the positive electrode was inserted between the two ears at the vertex in the skull. Earphones positioned in each ear provided a source for sound impulses. The acoustic stimuli were applied as tone bursts at 8, 16, and 32 kHz with a 5-ms plateau and 1-ms rise/fall time at a rate of 5/s. The impulse intensity ranged from 10 dB sound pressure level (SPL) to 90 dB SPL, with 10 dB increments. ABR threshold was defined as the lowest intensity capable of evoking a reproducible and visually detectable response of wave II/III complex. ABRs were recorded from wild-type (*RGS17* ^+/+^), inducible hair cell-specific RGS17 knockdown (*RGS17* ^+/−^), and inducible hair cell-specific RGS17 knockout (RGS17^−/−^) mice after one and two cycles of vehicle or cisplatin treatment.

### Cochlear whole-mount preparation

Cochleae were extracted from the temporal bone and perfused with 4% paraformaldehyde through the oval and round windows. They were then stored overnight at 4°C in the same solution. The cochleae were decalcified in 120 mM EDTA for 72 h at room temperature with constant stirring, and the EDTA solution was changed every 24 h. After decalcification, they were washed three times in PBS for 5 min each. Microdissection was then performed to isolate the cochlear turns (base, middle, and apex) for immunohistochemistry.

To prepare mid-modiolar sections, cochleae were removed from the EDTA solution and washed thrice in PBS for 5 min each. The cochleae were then submerged in 10% of sucrose with gentle rotation at room temperature for 30 min. Next, they were transferred to 15% sucrose and incubated at room temperature for 30 min before being moved to 4°C, where they were kept on rotation overnight. The cochleae were subsequently transferred to a 1:1 solution of 15% sucrose and OCT, incubated at 4°C overnight with rotation, and frozen at −80°C after being positioned in a cryomold.

### Hair cells and ribbon synapse count

Tile scans of cochlear whole mounts, apical, middle, and basal turns were imaged using a Zeiss confocal microscope. The same setting parameters were used for all images for counting OHCs and ribbon synapses. Myosin VIIa antibody was used to stain hair cells, whereas CtBP2 and GluR2 antibodies were used to stain presynaptic ribbons and postsynaptic glutamate receptors, respectively. The nucleus was stained with DAPI. Missing OHCs were manually counted in each cochlear turn using ×20 magnification, with the data presented as percentage loss relative to the total number of OHCs. Ribbon synapses from inner hair cells (IHCs) were imaged using ×60 magnification to include at least 12 IHCs per image. Functional (paired) ribbon synapses were defined as those possessing both CtBP2 + GluR2 immunolabeling apposing each other, whereas orphan (unpaired) synapses were defined as either CtBP2 or GluR2 puncta alone. Ribbon synapses were counted manually from three random microscopic fields for each turn and presented as the number of synapses per IHC.

### Immunohistochemistry

Immunolabeling for whole-mount and mid-modiolar sections was initiated by incubating the samples in ice-cold 100% methanol for 10 min at −20°C. Following this, the sections were blocked with blocking buffer (10% normal horse or goat serum, 1% BSA, and 1% Triton X-100) for 2 h at room temperature. The sections were then incubated with primary antibodies diluted with antibody dilution buffer as indicated in **Table 1** and incubated overnight at 4°C. On the next day, sections were washed thrice with 1× PBS and incubated with secondary antibodies as indicated in **Table 1**. Afterward, the sections were then counterstained with Hoechst (DAPI) (1:2,000) at room temperature for 20 min and mounted with Prolong^®^ Diamond Antifade Mountant (Invitrogen, Saint Louis, Missouri). All the sections were imaged using the same laser illumination settings for all the groups (*n* ≥ 6 cochleae/group). The tissue sections were imaged using a Zeiss LSM 800 confocal microscope (Zeiss Inc., USA). All the images were processed using Zen 2.5 (blue edition). The immunofluorescent intensity was measured using ImageJ software (version/Fiji). Briefly, intensity values were obtained by selecting the region of interest (ROI) for three samples per group. The nearest region to the ROI that exhibited no fluorescence was selected to determine the background intensity. This background value was subtracted from the ROI value to calculate the RGS17 intensity. The results were then presented as a percentage of the control group, with the control set at 100%.

**Table 1:** List of antibodies used in the experiments.

Antibody	Species, isotype	Dilution	Company (catalog #)
Myosin VIIa	Rabbit IgG	1:500	Thermo Fisher (PA1-936)
CtBP2	Mouse IgG1	1:500	BD Biosciences (612044)
GluR2	Mouse IgG2a	1:100	Millipore (MAB397)
CXCL1	Rabbit IgG	1:100	Abcam (AB86436)
CD45	Mouse IgG2a	1:100	Millipore (F10-89-4)
CD68	Mouse IgG1	1:100	Novus (NB600-985)
RGS17	Rabbit IgG	1:200	Novus(NBP180839)
Cleaved caspase-3	Rabbit IgG	1:200	Cell Signaling
Alexa Fluor™ 647 secondary antibody	Goat, IgG1	1:1,000	Thermo Fisher (A-21240)
Alexa Fluor™ 488 secondary antibody	Goat, IgG2a	1:1,000	Thermo Fisher (A-21131)
Rhodamine (TRITC) secondary antibody	Monkey, IgG	1:500	Jackson ImmunoResearch (711-025-152)
Alexa Fluor™ 647 secondary antibody	Goat, IgG	1:500	Thermo Fisher (A-21244)

CtBP2, C-terminal binding protein 2; GluR2, glutamate receptor 2; CXCL1, chemokine (C-X-C motif) ligand 1; CD, cluster of differentiation; RGS17, regulator of G protein signaling 17; IgG, immunoglobulin G.

### Statistics

Data are presented as mean ± standard error of the mean (SEM). Statistical significance of differences among groups used one-way analysis of variance (ANOVA) depending on the experiments, followed by Bonferroni’s multiple comparison test using GraphPad Prism version 6.07 for Windows. *P*-value <0.05 was considered significant.

## Results

### Hair cell-specific deletion of *RGS17* validation

The deletion of *RGS17* in the hair cells was evaluated by immunolabeling of hair cells in the organ of Corti with the anti-RGS17 antibody. The deletion of *RGS17* in the inducible hair cell-specific RGS17 knockout was verified by the absence of RGS17 immunolabeling in the cochlear hair cells, while the wild type showed immunolabeling of hair cells with anti-RGS17 (**Figure 1B**). We have designated the wild-type mice as (RGS17^+/+^), the inducible hair cell-specific RGS17 knockdown mice as heterozygous (RGS17^+/−^), and the inducible hair cell-specific RGS17 knockout mice as homozygous (RGS17^−/−^). We did not use globally manipulated RGS17 genetic mouse strains. All the mice described in the manuscript were either inducible hair cell-specific RGS17 knockdown (RGS17^+/−^), knockout (RGS17^−/−^), or wild type (RGS17^+/+^).

### Cisplatin induces RGS17 expression

**Figure 2:**
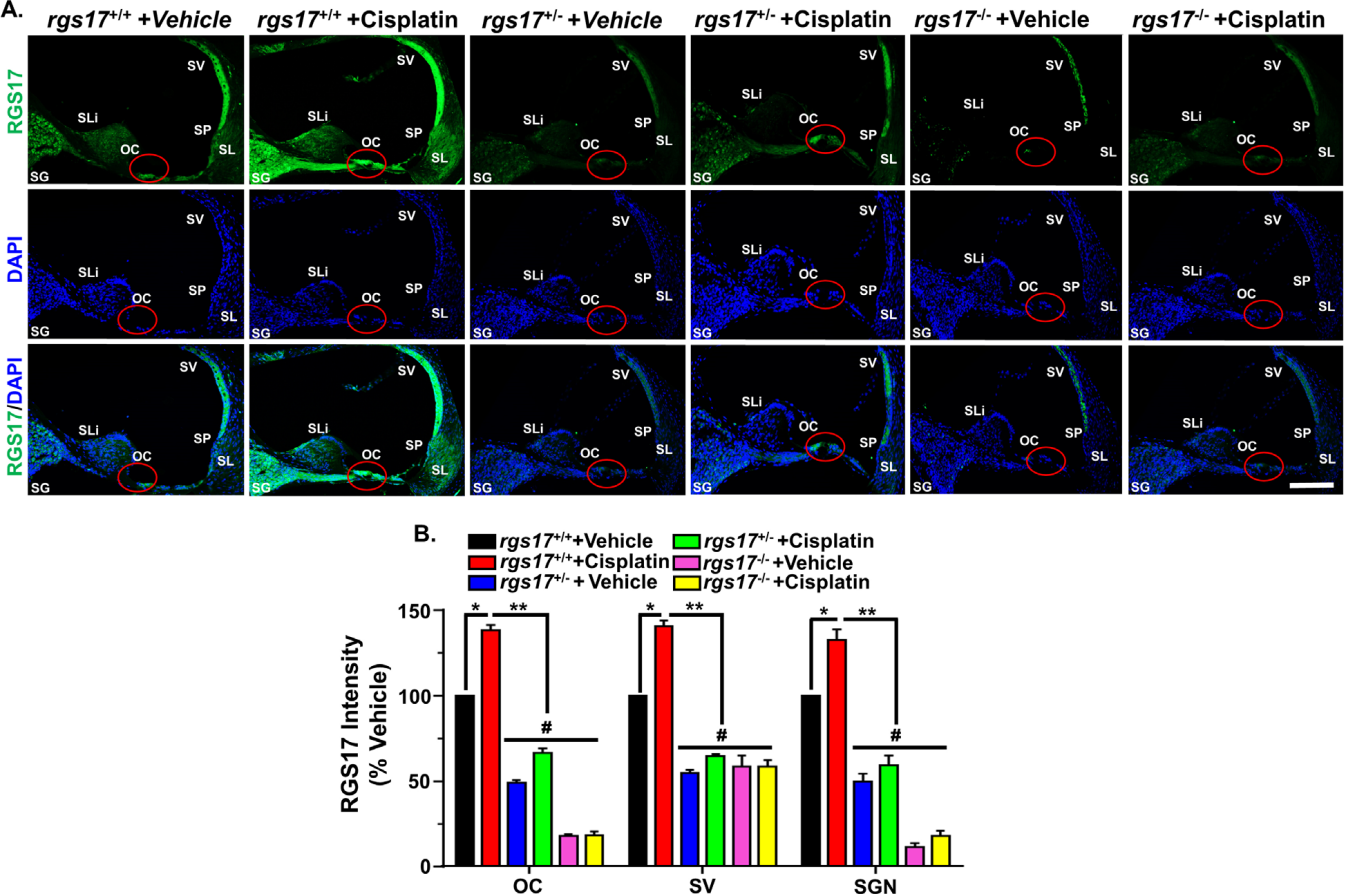
Cisplatin administration elevated RGS17 protein level in mid-modiolar sections. **(A)** Mice were treated with PBS or cisplatin (3.5 mg/kg IP) for two cycles. At the end of the second cycle, cochleae were harvested and processed for mid-modiolar sectioning. Cochlear sections were immunolabeled with RGS17 (green) and DAPI (blue). RGS17 wild-type mice (RGS17^+/+^) treated with cisplatin showed a higher level of RGS17 immunolabeling in the OC, SV, and SGN as compared to RGS17^+/+^ mice treated with vehicle (PBS). Inducible hair cell-specific RGS17 knockdown (RGS17^+/−^) mice showed slightly increased RGS17 immunolabeling in the OC, SV, and SGN after cisplatin treatment, while inducible hair cell-specific RGS17 knockdown (RGS17^−/−^) mice showed minimal RGS17 immunoreactivity in the OC, SV, and SGN after cisplatin treatment. Images are representative of six independent animals per treatment group. Red circles indicate the location of the organ of Corti. Scale bar = 50 µm. **(B)** Bar graph plotted from the data explained in **(A)** showing the average fluorescence intensities of RGS17 immunoreactivity in different regions of the cochlea (OC, SV, and SGN). Data represent mean ± SEM (*n* ≥ 6). (*) indicates significance (*P* < 0.05) compared to the vehicle group, while (**) indicates significance (*P* < 0.05) compared to cisplatin. (#) Indicates statistically significant differences (*P* < 0.05) from the cisplatin-treated group and the vehicle group (*n* = 4). Statistical analyses among groups were tested using one-way analysis of variance (ANOVA) followed by Bonferroni’s multiple comparison test.

Previously, our laboratory has shown that *RGS17* knockdown in the inner ear using siRNA attenuates cisplatin ototoxicity (18). To validate the integral role of RGS17 in cisplatin-induced hearing loss, wild-type mice, as well as inducible hair cell-specific *RGS17* knockdown (RGS17^+/−^) and inducible hair cell-specific *RGS17* knockout mice (RGS17^−/−^), were injected with cisplatin (3.5 mg/kg IP) or vehicle over two cycles (**Figure 1C**). RGS17 antibody was used to examine the expression of RGS17 in the hair cells. We observed increased RGS17 immunoreactivity in OHCs and IHCs in RGS17^+/+^ (wild-type) mice treated with cisplatin compared with RGS17^+/+^ treated with vehicle. Inducible hair cell-specific RGS17 knockdown (RGS17^+/−^) mice showed a slight increase of RGS17 immunolabeling in the hair cells after cisplatin injection, while no RGS17 immunolabeling was observed in the inducible hair cell-specific RGS17 knockout mice (*RGS17^−/−^*) following cisplatin administration (**Figure 1D**). In mid-modiolar sections, RGS17^+/+^ mice treated with cisplatin showed strong immunolabeling 2of RGS17 in the OC, SV, SGN, and type II spiral ligament compared to *RGS17^+/+^* treated only with vehicle. Hair cells heterozygous *RGS17^+/−^* mice showed a weak increase of RGS17 immunolabeling after cisplatin injection, while complete knockout of *RGS17^−/−^* gene in the hair cells showed no increase of RGS17 immunolabeling after cisplatin administration, the immunolabeling of RGS17 in *RGS17^−/−^* mice was similar to that observed in wild type treated with vehicle (**Figure 2A**). RGS17 immunofluorescence intensity for the OC, SV, and SGN in mid-modiolar sections was quantified in *RGS17^+/+^* mice treated with vehicle and normalized to 100% (**Figure 2B**), while cisplatin administration significantly increased RGS17 fluorescence intensities in *RGS17^+/+^* mice to 138.2 ± 2.5, 140.5 ± 3.6, and 132.4 ± 6.0 in the OC, SV, and SGN, respectively. In inducible hair cell-specific RGS17 knockdown RGS17^+/−^ mice treated with vehicle, RGS17 fluorescence intensities were 48.8 ± 1.8, 54.5 ± 1.9, and 49.9 ± 4.4 in the OC, SV, and SGN, respectively, which increased in *RGS17^+/−^* mice following cisplatin administration to 66.3 ± 2.7, 64.5 ± 1.2, and 59.1 ± 5.7 in the OC, SV, and SGN, respectively. Meanwhile, inducible hair cell-specific RGS17 knockout (*RGS17^−/−^*) mice were significantly protected against cisplatin-induced RGS17 expression, as detected by immunolabeling. The fluorescence intensities of RGS17 in homozygous RGS17^−/−^ mice treated with cisplatin were 18.3 ± 2.0, 58.3 ± 4.1, and 17.5 ± 3.2 in the OC, SV, and SGN, respectively, compared to RGS17 fluorescence intensities in RGS17^−/−^ mice treated with vehicle which were much lower at 17.9 ± 0.8, 58.5 ± 6.3, and 11.4 ± 2.2 in the OC, SV, and SGN, respectively (**Figures 2A, B**). The fluorescence intensity in the organ of Corti in homozygous RGS17^−/−^ likely reflects RGS17 immunolabeling of supporting Deiters cells (DCs) (**Figure 2B**). Higher magnification images of RGS17 in the OC, SV, and SGN are in the **Supplementary Figures S1A–C**. In the organ of Corti, wild-type RGS17^+/+^ mice treated with vehicle displayed immunolabeling in OHCs, IHCs, and DCs, which demonstrated the highest degree of RGS17 immunolabeling in the OHCs, IHCs, and DCs following two cycles of cisplatin injection; RGS17^+/−^ mice showed less expression; and RGS17^−/−^ mice showed the least or non-immunolabeling and therefore the greatest protection against cisplatin-induced RGS17 expression in OHCs, IHCs, and DCs (**Supplementary Figure S1A**). Similar trends were observed in the SV and SGN for RGS17^+/+^, RGS17^+/−^, and RGS17^−/−^ mice (**Supplementary Figures S1B, C**).

### RGS17 deletion attenuates cisplatin-mediated induction of immune cell markers

**Figure 3:**
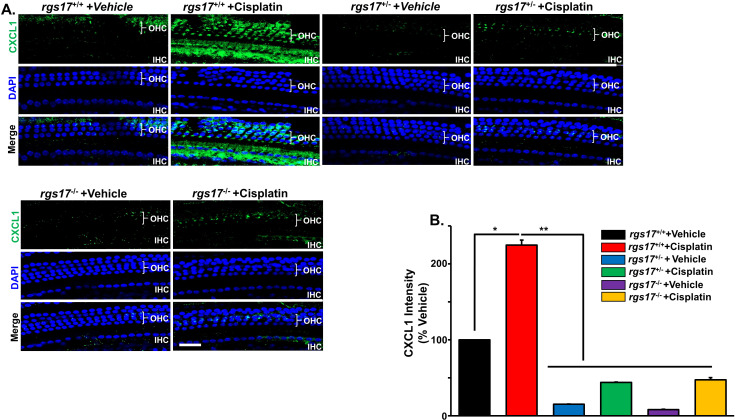
Effect of RGS17 deletion on cisplatin-induced CXCL1 in the hair cells. Mice were treated with PBS or cisplatin (3.5 mg/kg IP) for two cycles (**Figure 1C**). **(A)** Basal turns from each cochlea were immunolabeled with CXCL1 (green) and DAPI (blue). Wild-type RGS17 mice treated with cisplatin showed a higher level of CXCL1 immunoreactivity in the outer and inner hair cells as compared with wild-type mice treated with vehicle. Inducible hair cell-specific RGS17 knockdown (RGS17^+/−^) protects against cisplatin-induced CXCL1 immunoreactivity. Furthermore, inducible hair cell-specific RGS17 knockout (RGS17^−/−^) ameliorates cisplatin-increased CXCL1 in the outer and inner hair cells. Figures are representative of six independent animals per treatment group. Scale bar = 40 µm. **(B)** Protein expression quantity for CXCL1 was analyzed by ImageJ and presented as the normalized intensities versus vehicle-treated cochlea (*P* < 0.05, *n* = 4) using one-way ANOVA. Asterisk (*) indicates a statistically significant difference from vehicle; (**) indicates a statistically significant difference from cisplatin (*P* < 0.05, *n* = 4).

Previously, we have shown that CXCL1 increases in male Wister rat cochlea after cisplatin administration (11 mg/kg IP) (4). To test whether RGS17 regulates CXCL1 expression, wild-type RGS17^+/+^, heterozygous (RGS17^+/−^), and homozygous (RGS17^−/−^) mice were treated with either vehicle or cisplatin (3.5 mg/kg) for two cycles (**Figure 1C**). The cochleae were then collected and processed for whole-mount dissection and immunolabeled with CXCL1 antibody. CXCL1 immunoreactivity was low in the OHCs of RGS17^+/+^ mice. However, cisplatin administration increased CXCL1 immunolabeling in the organ of Corti cells (OHCs and IHCs). CXCL1 immunolabeling was ameliorated in RGS17^+/−^ and RGS17^−/−^ mice following cisplatin injection (**Figure 3A**). Thus, depletion of *RGS17* prevented cisplatin-induced CXCL1 expression. The fluorescence intensity of CXCL1 immunolabeling was expressed as a percentage in the control, which was set at 100%. Cisplatin administration significantly increased CXCL1 fluorescence intensity in the hair cells of *RGS17* ^+/+^ mice (224.6 ± 6.1). However, in *RGS17* ^+/−^ and *RGS17* ^−/−^ mice, CXCL1 fluorescence intensity was not highly elevated following cisplatin treatment, remaining at 45.0 ± 0.4 and 47.4 ± 2.9, respectively, compared to untreated *RGS17* ^+/−^ and *RGS17* ^−/−^ mice where CXCL1 fluorescence intensity was 15.2 ± 0.4 and 8.1 ± 0.5, respectively (**Figure 3B**).

**Figure 4:**
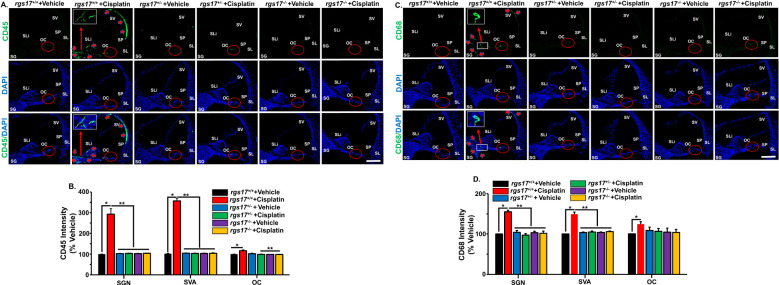
RGS17 knockout protects against cisplatin-induced inflammatory markers in the cochlea. Mice were treated with PBS or cisplatin (3.5 mg/kg IP) for two cycles. Cochleae were collected at the end of the second cycle and mid-modiolar sections were labeled with immune cell markers. **(A)** CD45 immunolabeling was observed in the SV, spiral limbus (SLi), OC, and surrounding the SGN in RGS17 wild-type mice treated with cisplatin. Heterozygous (RGS17^+/−^) and homozygous (RGS17^−/−^) mice treated with cisplatin showed no immunolabeling in the SV, SLi, and surrounding SGN. Red circles indicate the location of the organ of Corti. Red arrows indicate CD45 immunolabeling. Images are representative of six independent animals per treatment group. Scale bar = 50 µm. The magnification of the inset box is ×20 (20 µm). **(B)** Protein expression quantity for CD45 was analyzed by ImageJ and presented as the normalized intensities versus vehicle-treated cochlea using one-way ANOVA. Asterisk (*) indicates a statistically significant difference from vehicle; (**) indicates a statistically significant difference from cisplatin (*P* < 0.05, *n* = 4). **(C)** CD68 immunolabeling was observed in the SV, OC, and surrounding the SGN in RGS17 wild-type mice treated with cisplatin. Heterozygous RGS17 (RGS17^+/−^) and homozygous RGS17 (RGS17^−/−^) mice treated with cisplatin showed no immunolabeling in the SV, SLi, and surrounding the SGN. Red circles indicate the location of the organ of Corti. Red arrows indicate CD68 immunolabeling. Images are representative of six independent animals per treatment group. Scale bar = 50 µm. The magnification of the inset box is ×20 (20 µm). **(D)** Protein expression quantity for CD68 was analyzed by ImageJ and presented as the normalized intensities versus vehicle-treated cochlea using one-way ANOVA. Asterisk (*) indicates a statistically significant difference from vehicle; (**) indicates a statistically significant difference from cisplatin (*P* < 0.05, *n* = 4).

CD45 (leukocyte common antigen) is a receptor-linked tyrosine phosphatase present on white blood cells, playing an important role in the activation of T lymphocyte, neutrophil, and macrophage adhesion. We used a CD45 antibody to label the immune cells in the cochlea, as previously described (4). CD45 immunostaining was increased in the SGN and SV of RGS17^+/+^ mice treated with cisplatin, while CD45 immunolabeling was suppressed in both inducible hair cell-specific RGS17 heterozygous RGS17^+/−^ and homozygous RSG17^−/−^ mice after cisplatin injection for two cycles (**Figure 4A**). The fluorescence intensities of CD45 immunolabeling in RGS17^+/+^ mice significantly increased following cisplatin administration, reaching 293 ± 25.8, 357 ± 10.8, and 116.4 ± 4.1 in the SGN, SVA, and OC, respectively, compared to the untreated wild-type group, where CD45 fluorescence intensities were normalized to 100%. The fluorescence intensities of CD45 in *RGS17^+/−^* mice were not induced following cisplatin treatment, remaining at 102.8 ± 1.7, 102.8 ± 1.8, and 99.8 ± 1.4 in the SGN, SVA, and OC, respectively. This was consistent with untreated *RGS17* ^+/−^ where CD45 fluorescence intensities were 102.3 ± 1.5, 103.3 ± 1.25, and 101.9 ± 2.3 in the SGN, SVA, and OC, respectively. Similarly, CD45 fluorescence intensities in *RGS17^−/−^* mice were not induced following cisplatin treatment, remaining at 103.4 ± 1.5, 103.5 ± 1.5, and 98.9 ± 0.8 in the SGN, SVA, and OC, respectively. This was compared to untreated *RGS17* ^+/−^, where CD45 fluorescence intensities were 102.3 ± 1.6, 103.0 ± 1.8, and 98.3 ± 1.2 in the SGN, SVA, and OC, respectively (**Figure 4B**).

We also observed an increase in CD68 immunolabeling in the SGN and SV of RGS17^+/+^ mice treated with cisplatin. However, both inducible hair cell-specific RGS17 heterozygous RGS17^+/−^ and homozygous RGS17^−/−^ mice showed no immunolabeling of the CD68 immune cell marker in the SGN after cisplatin (**Figure 4C**). The fluorescence intensities of CD68 immunolabeling in RGS17^+/+^ mice were significantly increased after cisplatin administration, measuring 155 ± 3.6, 149.1 ± 5.2, and 124.5 ± 6.2 in the SGN, SVA, and OC, respectively, compared to the untreated wild-type group, where CD68 fluorescence intensity was normalized to 100%. In contrast, the fluorescence intensities of CD68 in *RGS17^+/−^* mice were not increased following cisplatin treatment, remaining at 97.1 ± 3.9, 105 ± 2.6, and 106.5 ± 7.5 in the SGN, SVA, and OC, respectively, compared with untreated *RGS17* ^+/−^, which exhibited CD68 fluorescence intensities of 104.4 ± 4.4, 103.5 ± 1.3, and 108.8 ± 8.1 in the SGN, SVA, and OC, respectively. Similarly, the fluorescence intensities of CD68 in *RGS17^−/−^* mice were not induced after cisplatin treatment, remaining at 101.7 ± 5.5, 105.8 ± 1.6, and 103.9 ± 7.3 in the SGN, SVA, and OC, respectively. This was consistent with untreated *RGS17* ^+/−^ where CD68 fluorescence intensities were 103.3 ± 3.3, 103.9 ± 1.1, and 104.6 ± 9.9 in the SGN, SVA, and OC, respectively (**Figure 4D**). These data suggest that specific deletion of the *RGS17* gene in hair cells reduces cochlear proinflammatory CXCL1 levels and prevents the entry of CD45 and CD68-positive immune cells into the cochlea. Higher magnification images were shown in **Supplementary Figures S2A–C**, **S3A–C**.

### Hair cell-specific deletion of RGS17 protects against cisplatin-induced hearing loss

**Figure 5:**
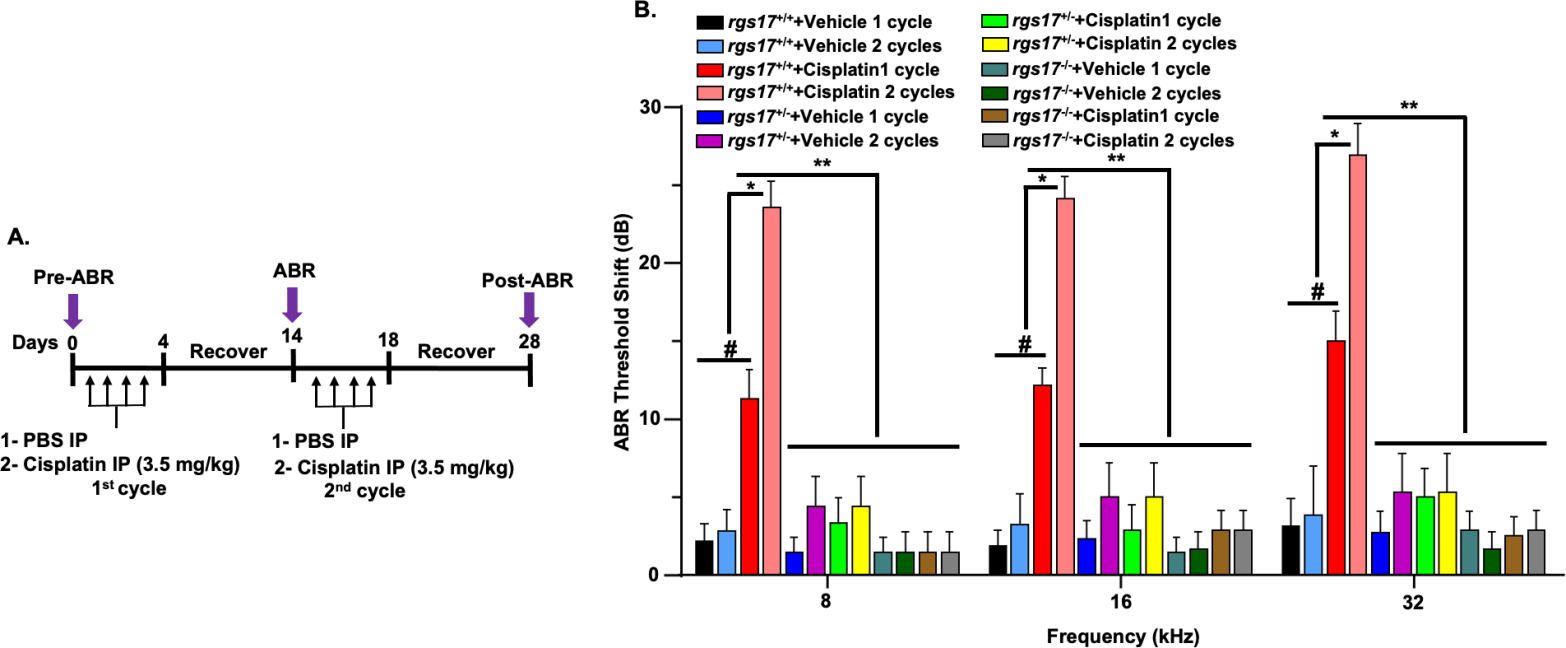
Partial knockdown or complete knockout of RGS17 rescued against cisplatin-induced ABR threshold. **(A)** Schematic of the experimental protocol that demonstrates the dosage and route of cisplatin. Pretreatment ABRs were recorded prior to cisplatin administration on RGS17 wild-type, knockdown, and knockout mice. Mice were administered either control or cisplatin (3.5 mg/kg) for two cycles, and each cycle consists of a 4-day cisplatin administration followed by 10 days of recovery. Post-treatment ABRs were assessed after each cycle of cisplatin injection (days 14 and 28). **(B)** ABR thresholds were increased significantly at 8, 16, and 32 kHz frequencies following one or two cycles of cisplatin injection. Knockdown of RGS17 (heterozygous RGS17^+/−^) or knockout of RGS17 (homozygous RGS17^−/−^) significantly attenuated cisplatin-induced elevation in ABR threshold shifts for 8, 16, and 32 kHz frequencies. Data represent mean ± SEM of eight mice per group. (#) indicates significance (*P* < 0.05) of one-cycle cisplatin treatment compared to vehicle groups (*n* = 8); (*) indicates significant difference (*P* < 0.05) of two cycles of cisplatin compared to one-cycle cisplatin and vehicle groups; (**) indicates significance (*P* < 0.05) between RGS17^+/−^ and RGS17^−/−^ treated with cisplatin or PBS from one or two cycles of cisplatin treatment.

To assess whether *RGS17* gene deletion in hair cells protects against cisplatin-induced hearing loss, we performed a functional study comparing wild-type mice with inducible hair cell-specific RGS17 knockdown (RGS17^+/−^) and knockout (RGS17^−/−^) mice. All genotypes were treated with either vehicle or cisplatin. A 28-day protocol was used to establish two cycles of cisplatin administration and recovery periods (**Figure 5A**). Pretreatment ABRs were recorded for all mice followed by intraperitoneal injection of either vehicle or cisplatin (3.5 mg/kg) for two cycles. Each cycle comprised 4-day injections followed by a 10-day recovery period. Pre-ABRs were recorded on day 0, while post-ABRs were recorded on day 14 (after the first cycle) and day 28 (after the second cycle, **Figure 5A**). Administration of the vehicle produced slight changes in ABRs, but there was no significant difference compared to pretreatment ABRs. RGS17^+/+^ mice showed a significant increase in ABR threshold shifts after the first cycle of cisplatin (11.2 ± 1.9, 12.1 ± 1.1, and 15.0 ± 1.9 dB at 8, 16, and 32 kHz, respectively). Similarly, after two cycles of cisplatin injection, ABR threshold shifts in wild-type RGS17^+/+^ mice were increased to 23 ± 1.6, 24 ± 1.3, and 26.9 ± 2.0 dB at 8, 16, and 32 kHz, respectively. Meanwhile, inducible hair cell-specific RGS17 knockdown (RGS17^+/−^) mice showed smaller ABR threshold shifts at all frequencies tested after cisplatin first cycle, with these values being 3.3 ± 1.5, 2.8 ± 1.6, and 5.0 ± 1.8, respectively, compared to 1.4 ± 0.9, 2.3 ± 1.1, and 2.7 ± 1.3, for RGS17^+/−^ vehicle-treated mice. ABR thresholds were elevated in RGS17^+/−^ mice treated with vehicle for two cycles, but these were close to 5 dB at all frequencies tested 4.3 ± 1.9, 5.0 ± 2.1, and 5.3 ± 2.4 dB, respectively. No increase in ABR threshold was observed for RGS17^+/−^ mice treated with cisplatin for two cycles, with thresholds measured at 4.3 ± 1.9, 5.0 ± 2.1, and 5.3 ± 2.4dB, respectively. This suggests that partial deletion of RGS17 significantly decreases cisplatin-induced ABR threshold shifts (**Figure 5B**). ABRs were also analyzed in inducible hair cell-specific RGS17 knockout (RGS17^−/−^) mice treated with either vehicle or cisplatin. ABR thresholds in RGS17^−/−^ mice treated with vehicle or cisplatin remained the same at 8 kHz for both cycles (1.4 ± 1.3 dB). For 16 kHz, the ABR thresholds for homozygous RGS17^−/−^ mice treated with two cisplatin cycles were 2.8 ± 1.3 and 2.8 ± 1.3 dB, respectively, compared to the RGS17^−/−^ mice treated with vehicle, which had thresholds of 1.4 ± 1.0 and 1.6 ± 1.0 dB, respectively. Also, cisplatin-induced alteration in hearing threshold at 32 kHz was abolished by complete knockout of RGS17 in the hair cells, with ABR threshold shifts remaining below 5 dB in both the vehicle- and cisplatin-treated groups (**Figure 5B**). These data suggest that inducible hair cell-specific RGS17 knockout (RGS17^−/−^) mice exhibit protective effects against cisplatin-induced ABR threshold. No significant ABR threshold shifts were observed among untreated groups, which included wild-type RGS17^+/+^, heterozygous RGS17^+/−^, and homozygous RGS17^−/−^ mice. Heterozygous RGS17^+/−^ mice treated with one cycle (blue bars) or two cycles (purple bars) of vehicle showed no significant ABR threshold shifts compared to their respective untreated wild-type (RGS17^+/+^) mice at 8, 16, and 32 kHz. A similar trend was observed in homozygous *RGS17* ^−/−^ mutant mice, where those treated with one cycle (deep turquoise bars) or two cycles (deep green bars) of vehicle showed no significant ABR threshold shifts compared to their respective untreated wild-type (RGS17^+/+^) mice at 8, 16, and 32 kHz. This suggested that RGS17 is a non-factor for the survival or function of hair cells, and the mutant mice were not deaf prior to cisplatin treatments (**Figure 5B**).

**Figure 6:**
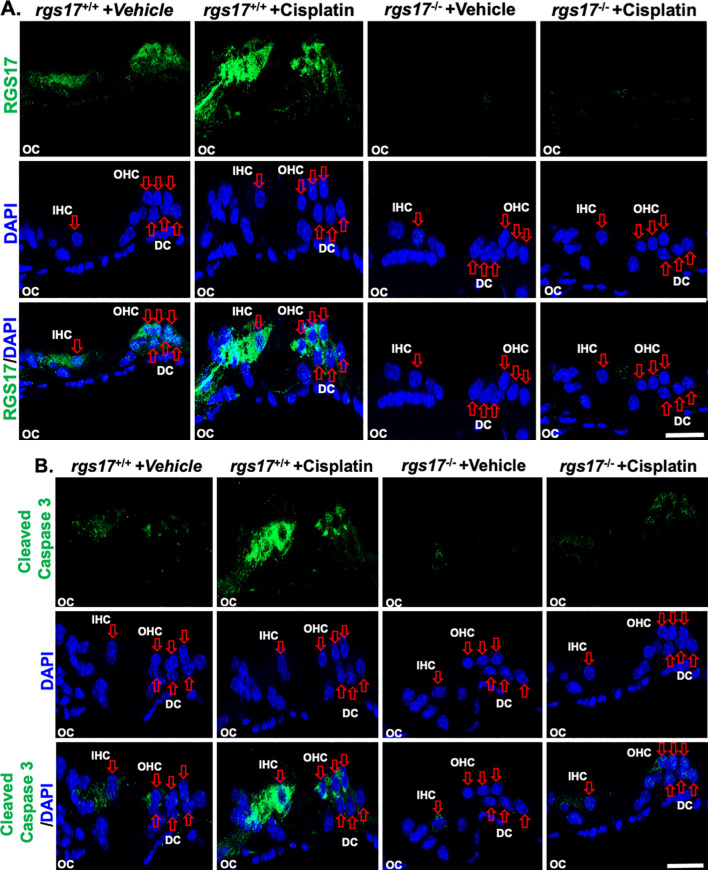
Cisplatin administration increased RGS17 and cleaved caspase-3 protein levels in cochlear mid-modiolar sections. Mid-modiolar sections from mice treated with one cycle of PBS or cisplatin (3.5 mg/kg) were immunolabeled with RGS17, cleaved caspase-3 (green), and DAPI (blue). Sections were captured at high magnification to distinguish the RGS17 and cleaved caspase-3 intensity in the organ of Corti parts. Immunolabeling of RGS17 and cleaved caspase was increased in RGS17 wild-type mice (RGS17^+/+^) treated with cisplatin compared to the control group, while knockout of the RGS17 gene ameliorated cisplatin-induced RGS17 **(A)** and cleaved caspase-3 **(B)** immunolabeling in the organ of Corti. Images collected from six independent animals per treatment group. Images are representative of six independent animals per treatment group. Scale bar = 20 µm.

To establish a temporal relationship between RGS17 expression and auditory cell apoptosis, expression of RGS17 and cleaved caspase-3 was measured and compared between RGS17 wild-type and knockout animals. After one cycle of cisplatin treatment, RGS17 wild-type mice showed an increased expression of RGS17 and caspase-3, while inducible hair cell-specific RGS17 knockout mice showed no change in RGS17 or caspase-3 expression (**Figures 6A, B**). This suggests that the expression of RGS17 has a temporal relationship with cleaved caspase-3 and the apoptotic pathway. By knocking out *RGS17*, the caspase-3 apoptotic pathway may be circumvented, thereby protecting auditory cells from apoptosis and reducing cisplatin ototoxicity.

**Figure 7:**
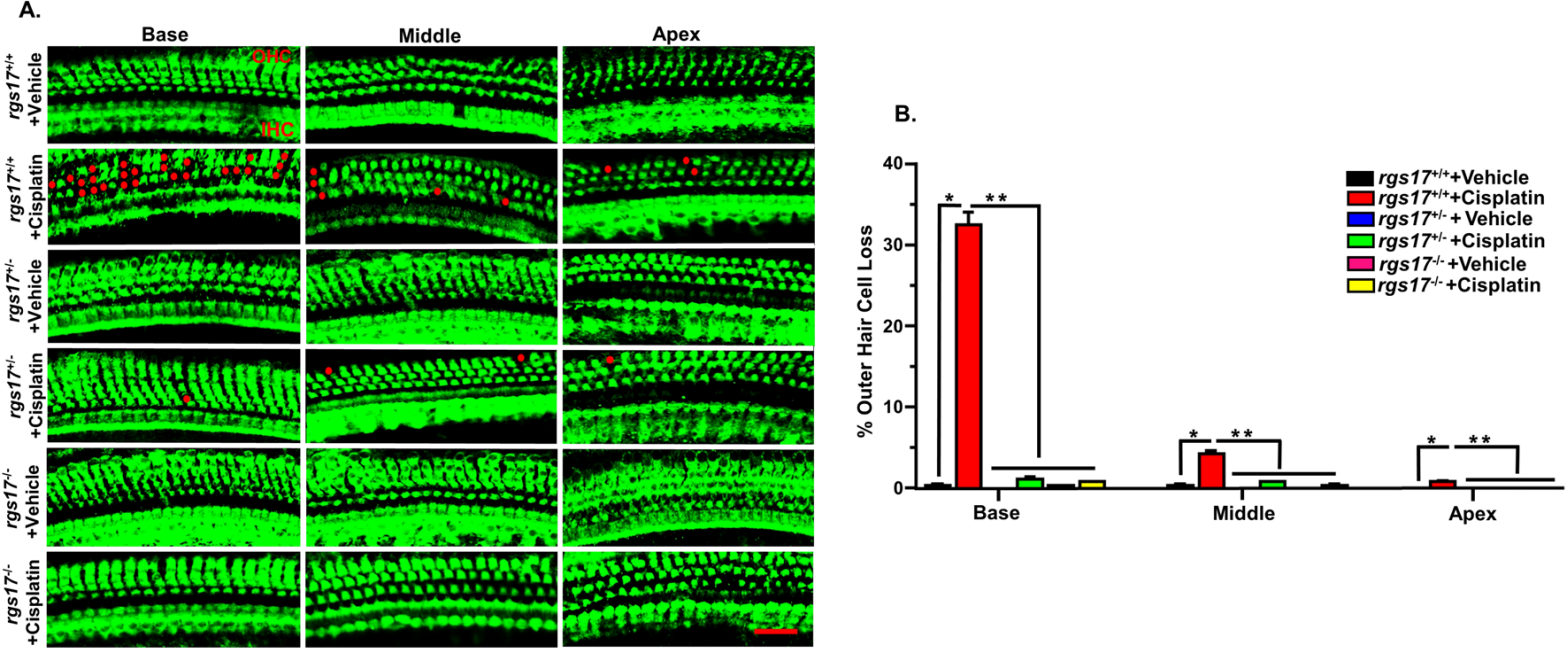
Protection against cisplatin-induced OHC loss in mice with partial knockdown of RGS17^+/−^ and complete knockout of RGS17^−/−^. Mice were treated with PBS or cisplatin (3.5 mg/kg IP) for two cycles. At the end of the second cycle, the cochleae were processed for whole-mount dissection to isolate the three different turns (apex, middle, and base). **(A)** Sections were stained for myosin VIIa (green). Representative images show significant OHC damage (red dots) in RGS17 wild type following cisplatin treatment, while heterozygous RGS17^+/−^ and homozygous RGS17^−/−^ mice were protected against cisplatin-induced OHC loss. **(B)** The bar graph represents the missing OHCs in the basal, middle, and apex turns of the cochlea presented in **(A)**. Data are presented as the mean ± SEM. (*) indicates a significant difference (*P* < 0.05) from the vehicle group, while (**) indicates a significant difference (*P* < 0.05) from the cisplatin (*n* = 6)-treated group. Statistical analyses among groups were tested using one-way analysis of variance (ANOVA). Scale bar = 40 µm.

We also investigated whether RGS17 is implicated in cisplatin-induced OHC loss. Using two cycles of cisplatin injection protocol (**Figure 5A**), cochleae were collected for whole-mount dissection. Manual counting of OHCs in wild-type RGS17^+/+^ mice showed an increase in OHC loss after cisplatin treatment, with percentage losses at 0.8% ± 0.0, 4.1% ± 0.4 and 32.5% ± 1.5 in the apical, middle, and basal turns, respectively. However, RGS17^+/−^ mice showed significant protection against cisplatin-induced OHC loss, limiting OHC loss to 0.0% ± 0.0, 0.8% ± 0.0, and 1.1% ± 0.2 from these respective regions, without change in IHC number. Following a similar trend, homozygous RGS17^−/−^ mice reduced the effect of cisplatin-induced OHC loss, with OHC loss limited to 0.0% ± 0.0, 0.2% ± 0.2, and 0.8% ± 0.0 from these respective regions. These results suggest that inducible hair cell-specific RGS17 reduction (RGS17^+/−^) or depletion (RGS17^−/−^) can reduce OHC loss after two cycles of cisplatin treatment (**Figures 7A, B**).

We also examined the missing OHCs after one cycle of cisplatin administration. OHC counting from wild-type RGS17^+/+^ mice treated with cisplatin showed 3.6% ± 0.2 and 26.1% ± 0.3 loss from the middle and basal turns, respectively, while heterozygous RGS17^+/−^ significantly reduced the effect of cisplatin to 0.8% ± 0.0 and 1.1% ± 0.2 of OHC loss, without altering the IHC number. Similarly, complete knockout of the RGS17^−/−^ gene decreased the effect of cisplatin, limiting OHC loss to 0.3% ± 0.2 and 0.3% ± 0.2 from these respective regions (**Supplementary Figures S4A, B**).

### Knockout of RGS17 in the hair cells reduces cisplatin-induced synaptopathy

**Figure 8:**
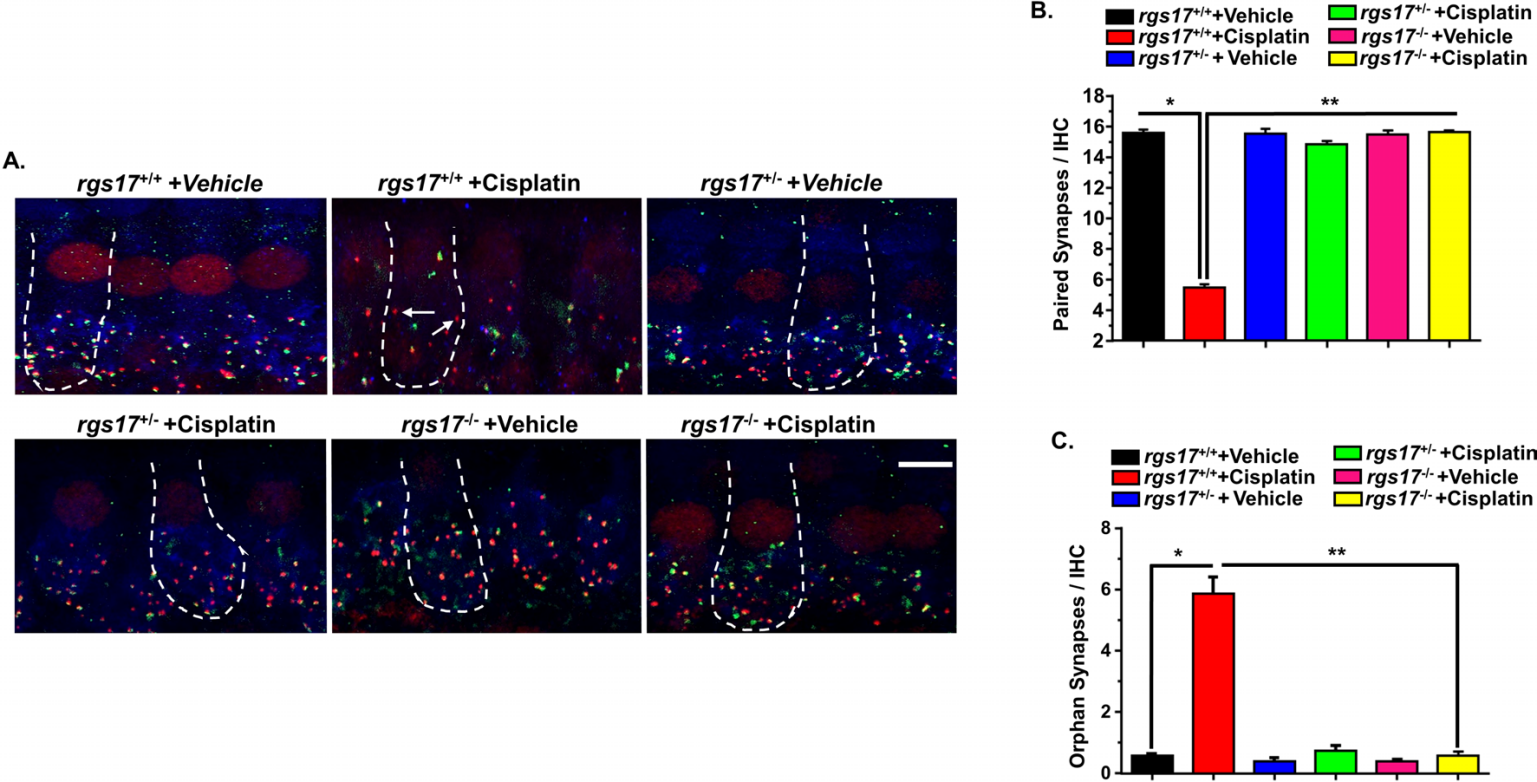
Protection against cisplatin-induced synaptopathy in mice with partial knockdown of RGS17^+/−^ and complete knockout of RGS17^−/−^. Whole-mount sections for PBS or cisplatin (3.5 mg/kg)-treated mice for two cycles were stained with myosin VIIa (blue), CtBP2 presynaptic marker (red), and GluR2 postsynaptic marker (green). **(A)** Whole-mount images from the basal turn indicated that ribbon synapse per IHC was decreased in RGS17 wild-type mice following two cycles of cisplatin treatment, whereas these effects were abolished by knockdown or complete knockout of the RGS17 gene in hair cells. Orphan synapses were represented as staining with either GluR2 or CtBP2 alone, but not both (indicated by white arrows). **(B)** The graph represents the number of synaptic ribbons per IHC, which were preserved by inducible hair cell-specific RGS17 knockdown and knockout. **(C)** Cisplatin treatment for two cycles significantly induced orphan synapses per IHC which were abolished by inducible hair cell-specific RGS17 knockdown and knockout. Data are presented as the mean ± SEM of six animals. Asterisk (*) indicates a significant difference from the vehicle group, while (**) indicates a significant difference from the cisplatin group. Scale bar = 10 µm. The magnification of the inset box is ×20 (20 µm).

Ototoxic drugs, aging, and noise are implicated in the loss of ribbon synapses and SGN (35–39). Cochlear synaptopathy decreases supra-threshold ABR wave I amplitudes and increases latencies as a result of losing synapses between type I SG afferent neurons and IHCs (40). Our laboratory has previously shown that cochlear synaptopathy occurs subsequent to cisplatin treatment (4). To assess synapse integrity, we labeled the presynaptic ribbons with an antibody against C-terminal-binding protein 2 (CtBP2) and the postsynaptic terminals with an antibody against glutamate receptor (GluR2). IHCs were labeled with antibodies against myosin VIIa (blue). Synapses were confirmed by immunolabeling whole-mount sections with these antibodies. Paired synapses were detected as yellow fluorescence [a combination of red (CtBP2) plus green (GluR2)]. We evaluated IHC-synapse for two cycles of cisplatin injection protocol (**Figure 5A**). The average of paired synapses showing both CtBP2 and GluR2 staining per IHC in the basal turn of wild-type RGS17^+/+^ mice treated with vehicle was 15.6 ± 0.2, and this number significantly decreased to 5.4 ± 0.2 following two cycles of cisplatin treatment in wild-type RGS17^+/+^. Paired synapses were significantly rescued in inducible hair cell-specific RGS17 knockdown (heterozygous, RGS17^+/−^) mice after two cycles of cisplatin treatment, with an average of 15.2 ± 0.2. Similarly, inducible hair cell-specific RGS17 depletion (homozygous, RGS17^−/−^) significantly prevented the loss of paired synapses after two cycles of cisplatin treatment, reducing the loss to 15.4 ± 0.1 (**Figures 8A, B**). The number of orphan synapses after CtBP2 or GluR2 staining not paired with each other increased from 0.5 ± 0.1 per IHC in wild-type RGS17^+/+^ mice treated with vehicle to 5.8 ± 0.5 in wild-type RGS17^+/+^ treated with two cycles of cisplatin. Both inducible hair cell-specific RGS17 knockdown and knockout protected against cisplatin-increased orphan synapses, which averaged 0.7 ± 0.1 and 0.5 ± 0.1, respectively (**Figure 8C**).

## Discussion

The current study demonstrates that RGS17 is upregulated in the cochlea following cisplatin injection. RGS17 likely plays a role in cochlear inflammation and is implicated in cisplatin-mediated hearing loss. On the other hand, depletion of RGS17 protects the cochlea against cisplatin-mediated inflammation and loss of OHC and IHC synaptopathy. RGS17 seems to play an important role in cochlear inflammation, as depletion of this gene mitigates the expression of proinflammatory markers such as CXCL1 and decreases the levels of CD45 and CD68-positive immune cells in the cochlea, while cisplatin-mediated activation of RGS17 upregulates the production of CXCL1 and increases both CD45 and CD68-positive immune cells in the cochlea. Collectively, these studies highlight the RGS17/CXCL1 pathway as a mediator of cisplatin ototoxicity. Thus, inhibiting RGS17 could represent a novel approach for reducing cisplatin-induced cochlear toxicity.

Previously, we have shown that cisplatin upregulates *RGS17* and *CXCL1* genes in the cochlea (4, 18). In this study, the *RGS17* tissue-specific knockout mouse model was developed and used to examine how depletion of RGS17 influences CXCL1-mediated cochlear inflammation.

G proteins and GPCRs are involved in many physiological functions, including immune responses and neurotransmission. Their dysfunction is linked to various diseases like immune system disorder and cardiovascular and Alzheimer’s diseases, making them key targets in drug development. G proteins like G_i_, G_o_, G_s_, G_q_, and G_z_ are expressed throughout the cochlea and play an important role in GPCR-mediated signaling within auditory sensory structures. GPCRs regulate the activation and inactivation of G proteins. (21, 23−25, 41, 42) Activation of GPCR modulates G protein by catalyzing the exchange of GDP for GTP on the α subunit in the G protein, leading to dissociation of the ability of G_α_-GTP to modulate activity of effector proteins such as adenylyl cyclase, phosphatases, voltage-dependent Ca^2+^ channel, and phospholipase-C (43). GTPase-mediated hydrolysis of GTP to GDP effectively turns off G_α_ signaling. GPCRs recognized by RGS proteins include D_2_ and D_3_ dopaminergic (44), M_1_ and M_2_ muscarinic pre- and postsynaptic (45, 46), and α_1A_-, β_1_-, and β_2_-adrenergic (47–49) receptors. RGS17 binds to and elevates the GTPase activity of G_α_ subunits, including G_αi1–3_, G_αz_, G_αq_, and G_αo_ (50). RGS17 acts as a GTPase-activating protein (GAP) by attenuating G_αi/o_-mediated cAMP formation which results in the upregulation of protein kinase A signals that promote cancer proliferation, migration, and invasion (30, 33, 51). Agents that increase cAMP are anti-inflammatory and inhibit neutrophil migration (52). Since RGS17 decreases cAMP formation, we hypothesize that cisplatin-driven overexpression of RGS17 may exert a similar action at the level of G_α_ to promote inflammation, leading to cochlear damage and hearing loss.

A non-canonical pathway for RGS17 in the brain is exhibited by its ability to interact with the histidine triad nucleotide-binding protein 1 (HINT1) complex. The HINT1–RGS17 complex couples mu opioid receptors to protein kinase C-γ and assists in the activation of the ERK–MAP kinase pathway (53). This mechanism is instrumental in desensitization of the CB2R response to endocannabinoids. In the cochlea, not only CB2R is expressed in the OC, SV, and SG neurons, but also downregulation of CB2R occurs subsequent to cisplatin exposure (19). Cisplatin clearly induces the expression of RGS17 in many parts of the cochlea, and its overexpression induces hearing loss. In contrast, knockdown or knockout of RGS17 protects against cisplatin-induced hearing loss. RGS17 activity imitates cisplatin-induced hearing loss by increasing the ABR threshold and producing cochlear synaptopathy. Previously, we have shown that activating GPCRs (by CB2R agonists) in the cochlea protects against cisplatin-induced hearing loss (18, 19). Therefore, the upregulation of RGS17 by cisplatin could antagonize the otoprotective activity of CB2R.

CXCL1 is the major chemoattractant involved in the recruitment of neutrophils to the site of injury, and the attracted neutrophils produce proinflammatory cytokines and proteases (54). Massive infiltration of neutrophils leads to uncontrolled production of cytokines such as CXCL1 which is considered dangerous and participates in tissue damage. (4, 55) CXCL1 expression in the OC and resident macrophages orchestrates signals for neutrophil migration into the cochlea from the peripheral circulation. Neutrophil migration may be mediated by CXCL1 which is expressed by fibroblasts, endothelial cells, pericytes, and spiral ganglion neurons (56). LPA administration provokes CXCL1 production and immune cell migration in the hippocampus, and spinal cord neuroinflammation associated with peripheral neuropathy involves CXCL1 (57). Prolonged or excessive expression of CXCL1 induces the accumulation of DAMP molecules at the trauma site, including high mobility group box 1 protein (HMGB1) and S100 proteins, which are released from damaged cells. HMGB1 proteins mediate inflammatory responses via interactions with surface receptors, such as receptors for advanced glycation end products (RAGE), TLR2, TLR4, nuclear factor kappa B (NF-κB), and STAT1 transcription factors (58). Previous studies from our lab demonstrate that STAT1 (35) and CXCL1 (4) are common mediators of cisplatin-induced inflammation. Cisplatin-induced ototoxicity involves the activation of proinflammatory cytokines through the NF-κB and CXCL1 pathways in auditory cells. During cisplatin treatment, CXCL1 signaling triggers stress pathways via NOX3 and iNOS activation, which can promote apoptosis and lead to the activation of cleaved caspase-3. RGS17 serves as a key modulator of stress pathways in the cochlea following cisplatin administration. Therefore, RGS17 knockout enhances cell survival in this stressful microenvironment, potentially preventing caspase-3 cleavage. Moreover, the elevation of RGS17 induced Ser^727^ STAT1 phosphorylation and reduced Tyr^701^ STAT3 phosphorylation (24, 36). Overexpression of *CXCL1* also increases STAT1 phosphorylation and decreases STAT3 phosphorylation, elevating the STAT1/STAT3 ratio which promotes cell apoptosis. On the other hand, increasing the STAT3/STAT1 ratio promotes survival homeostasis in the cochlea. Interestingly, the promoters of *RGS17* and *CXCL1* genes have STAT1-binding sites (18, 35, 59, 60). As a result, RGS17 can orchestrate proinflammatory mediators, which include CXCL1.

Tonic activation of chemokine receptors such as CXCR2 (GPCR) increases CD45 and CD68-positive immune cells in the cochlea. This activation is mediated by CXCL1 and IL-8 (4). Activation of CXCR2 by a chemoattractant (such as CXCL1) activates G proteins and promotes dissociation of the Gα_i_ and G_βγ_ complex (61). The α subunit decreases adenylyl cyclase activity which leads to decreased cAMP and cAMP-dependent protein kinase activity (62). RGS17 positively regulates the protein kinase via GAP activity exerted on the G_αi/o_ subunit. We speculate that RGS17 activation via cisplatin is one of the mechanisms associated with cochlear inflammation mediated by chemokine/chemokine receptors.

This study provides novel evidence that depletion of RGS17 ameliorates cisplatin ototoxicity. *RGS17* knockout prevents OHC damage induced by cisplatin. These otoprotective mechanisms are mediated via the reduction of inflammatory signals such as CXCL1, known to mediate cisplatin ototoxicity (4). Although the SV exhibited increased inflammation induced by cisplatin, the current study did not explore hearing loss resulting from stria dysfunction, which can be explored in future studies. It is not clear why specific knockout of RGS17 in the OC protects the spiral ganglion and stria vascularis from cisplatin-induced injury. Cisplatin-treated mice showed an increase in ROS and STAT1 transcription (63). This ultimately increases ROS production, promoting CXCL1 (4), STAT1 (35), neutrophil migration, hair cell apoptosis, and necrotic cell death (64). In preliminary studies, we demonstrate that the STAT1 inhibitor, EGCG, significantly decreases RGS17 expression (data not shown), highlighting a potential pathway linking ROS production to STAT1/CXCL1 expression. Depletion of RGS17 decreases ROS production (18) and, as a consequence, the production of CXCL1. Our findings support this proposed mechanism, as RGS17 depletion blocks CXCL1 production in hair cells and protects against cisplatin-induced hearing loss.

RGS17 appears to play a role in cisplatin-induced IHC synaptopathy. Previously, we have shown that EGCG, which possesses both antioxidant and anti-inflammatory properties, can protect against cisplatin-induced hearing loss and IHC synaptopathy (35). Noise-induced hearing loss (NIHL) damages the cochlear nerve and IHCs due to glutamate excitotoxicity (38). Glutamate agonists exacerbate injury of the cochlear nerve, whereas glutamate antagonist preserves cochlear neurons against excitotoxicity (65). Synaptopathy is dependent on different factors like TNF-α, transforming growth factor (TGF-β) (4), iNOS, IL-1β (66), and the α-amino-3-hydroxy-5-methyl-4-isoxazolepropionic acid (AMPA) receptor (67), while neurotrophin-3 (NT-3) and brain-derived neurotrophic factor (BDNF) preserve synaptic integrity. Noise or aminoglycosides produce a deficit in NT-3 and BDNF (68, 69). We postulate that cisplatin could reduce NT-3 and BDNF levels and thereby promote the loss of ribbon synapses. In the current study, overexpression of *RGS17* increases inflammatory markers such as CXCL1, while knockdown of *RGS17* decreases inflammation and preserves synaptic integrity. The finding that inhibition of RGS17 protected against synaptic loss suggests that inflammation mediated by RGS17 might reduce the levels of the trophic factors at these synapses.

**Figure 9:**
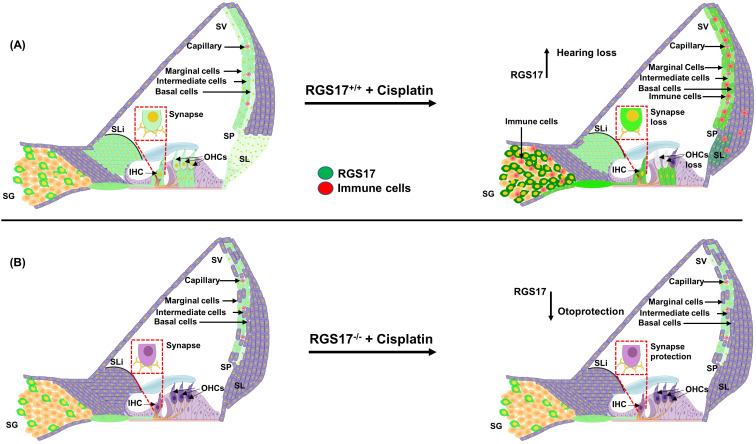
Hypothesis of cisplatin’s impact on cochlear inflammation via the RGS17 gene. **(A)** Cisplatin injection elevates the RGS17 level, especially in the SGN, SV, OC, and type II SL. Also, the recruitment of CD45 and CD68-positive immune cells and their migration to the cochlea through the spiral ligament and stria vascularis. **(B)** Hair cell-specific knockout of the RGS17 gene (RGS17^−/−^) followed by cisplatin injection downregulates RGS17 expression in the SGN, SV, and type II SL and inhibits the migration of CD45 and CD68-positive immune cells to the cochlea.

In summary, our study demonstrates that RGS17 plays a critical role in cochlear inflammation and cisplatin-induced hearing loss. Cisplatin administration induces RGS17, mainly in the spiral ganglion neuron, stria vascularis, and organ of Corti. Upregulation of RGS17 by cisplatin increases the number/expression of CD45 and CD68-positive immune cells in the cochlea. However, knockout or knockdown or inhibition of this gene protects against cisplatin-induced ototoxicity and inflammation (see **Figure 9**). Therefore, RGS17 could be a novel target for the amelioration of cisplatin-induced hearing loss.

## Data Availability

The original contributions presented in the study are included in the article/**Supplementary Material**. Further inquiries can be directed to the corresponding author.

## Supplementary material

Supplementary Figure 1 Cisplatin administration increased RGS17 protein level in cochlear mid-modiolar sections. Mid-modiolar sections from mice treated with PBS or cisplatin (3.5 mg/kg) for two cycles were immunolabeled with RGS17 (green) and DAPI (blue). Sections were captured at high magnification to distinguish the RGS17 intensity in cochlear mid-modiolar parts. **(A)** RGS17^+/+^ mice treated with cisplatin demonstrated higher level of RGS17 immunolabeling (see red arrow) in the in OHCs, IHC and supporting Deiters cells (DCs), whereas inducible hair cell-specific RGS17 knockdown/(RGS17^+/-^) ameliorated cisplatin induced RGS17 immunolabeling in OHCs, IHCs and DCs. Complete inducible hair cell-specific RGS17 knockout (RGS17^-/-^) indicates full protection against cisplatin induced RGS17 immunolabeling in OHCs, IHCs and DCs. **(B)** and **(C)** Immunolabeling of RGS17 was increased in RGS17 wild type mice (RGS17^+/+^) treated with cisplatin compared to control group, while partial knockdown or complete knockout of RGS17 gene ameliorated cisplatin induced RGS17 immunolabeling in SV and SGN. Images collected from six independent animals per treatment group. Images are representative of six independent animals per treatment group. Scale bar = 20 µm.

Supplementary Figure 2 RGS17 knockout abolishes cisplatin-induced CD45 positive immune cell in the cochlea. Mice were treated with PBS or cisplatin (3.5 mg/kg) for two cycles. At the end of second cycle, mice were sacrificed and cochlea were harvested, fixed with 4% PFA and decalcified for 72 h. then, processed for mid-modiolar sectioning. Cochlear sections were immunolabeled with CD45 (green) and DAPI (blue). Sections were captured at higher magnification to distinguish the CD45 immunolabeling. RGS17 wild type mice treated with cisplatin showed increased number of CD45 (see red arrow) positive immune cells in **(A)** SGN and **(B)** SV, whereas inducible hair cell-specific RGS17 knockdown and knockout reduced CD45 positive cells in SGN and SV. **(C)** No CD45 immunolabeling in the OC was detected following cisplatin treatment. Images were collected from six independent animals per treatment group. Scale bar = 20 µm. The magnification of inset box in 2A is 20X.

Supplementary Figure 3 RGS17 knockout inhibits cisplatin-induced CD68 positive immune cell in the cochlea. Mice were treated with PBS or cisplatin (3.5 mg/kg) for two cycles. At the end of second cycle, cochlea were collected, then, processed for mid-modiolar sectioning. Cochlear sections were immunolabeled with CD68 (green) and DAPI (blue). Sections were imaged at high magnification to show the CD68 immunolabeling. In SGN **(A)** and SV **(B)** CD68 positive cells (see red arrows) were increased following cisplatin injection in RGS17 wild type mice (RGS17^+/+^) treated with cisplatin, whereas inducible hair cell-specific RGS17 knockdown and knockout inhibits expression of CD68 positive cells in SGN and SV. **(C)** No CD68 immunolabeling in the organ of Corti was detected following cisplatin treatment. Images were collected from six independent animals per treatment group. Scale bar = 20 µm. The magnification of inset box in 3A is 20X.

Supplementary Figure 4 knockout of RGS17 protects against cisplatin-induced hearing loss. Cochlear whole mount dissection from mice treated with one cycle of PBS or cisplatin (3.5 mg/kg) were isolated **(A)** Sections were stained for myosin VIIa (green). Representative images show significant OHCs damage (red dots) in RGS17^+/+^ followed cisplatin treatment, while heterozygous RGS17^+/-^ and homozygous RGS17^-/-^ mice were protected against cisplatin-induced OHCs loss. **(B)** Bar graph represent the missing OHCs in basal and middle turns of cochlea presented in **(A)**. Apex showed no missing OHCs after cisplatin treatment in RGS17^+/+^ mice. Data are presented as the mean ± SEM. (*) indicates significant difference (p < 0.05) from vehicle group, while (**) indicate significant difference (p < 0.05) from cisplatin (n=6). Statistical analyses among groups were tested using one-way analysis of variance (ANOVA). Scale bar = 40 µm.
