# Supplementary figures and images for: Role of RGS17 in cisplatin-induced cochlear inflammation and ototoxicity via caspase-3 activation
Source: Front Immunol. 2025 Feb 21;16:1470625. doi: 10.3389/fimmu.2025.1470625 (PMC11885124; doi:10.3389/fimmu.2025.1470625)

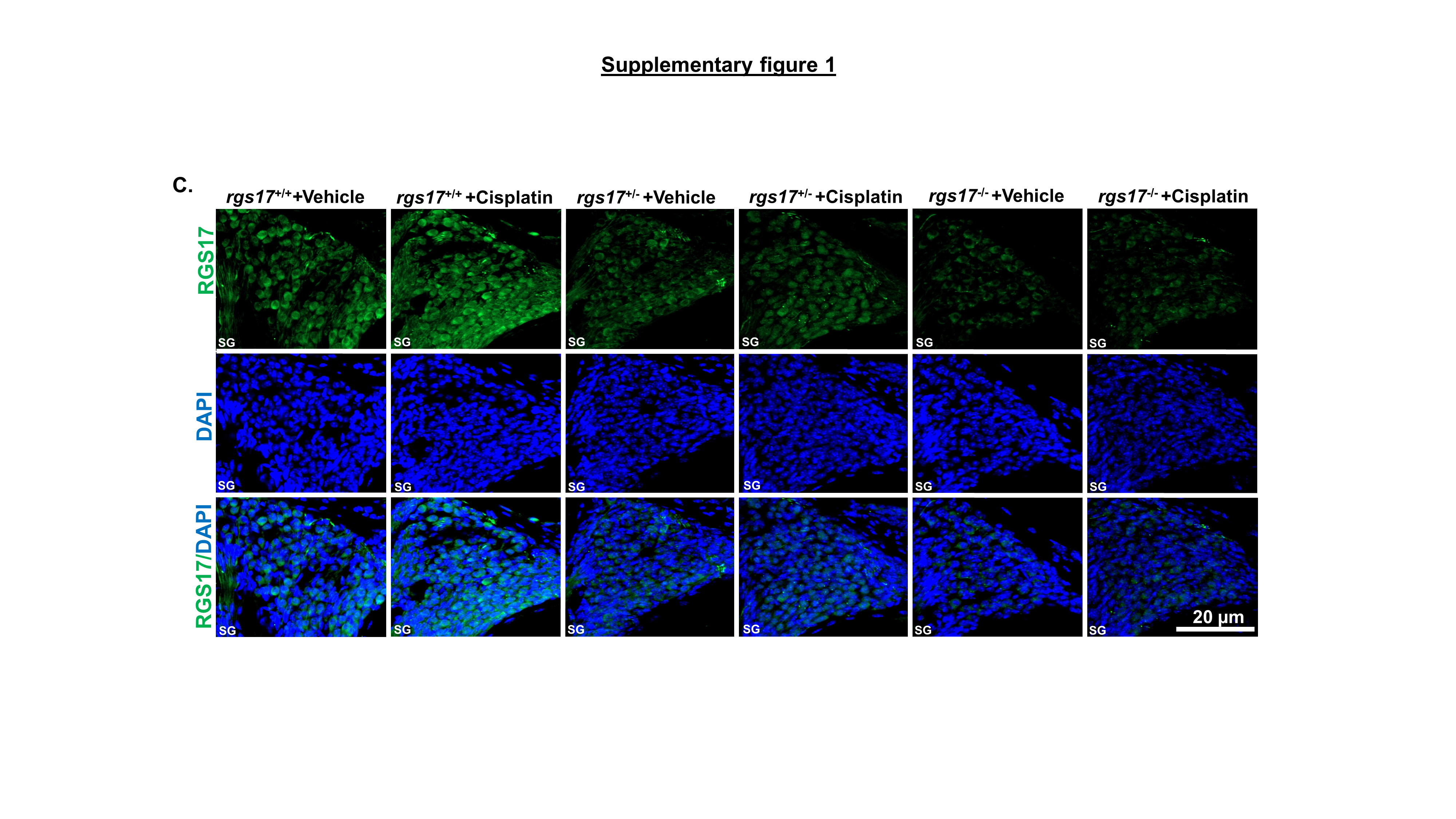

Supplement: Supplementary Figure 1 — Cisplatin administration increased RGS17 protein level in cochlear mid-modiolar sections. Mid-modiolar sections from mice treated with PBS or cisplatin (3.5 mg/kg) for two cycles were immunolabeled with RGS17 (green) and DAPI (blue). Sections were captured at high magnification to distinguish the RGS17 intensity in cochlear mid-modiolar parts. (A) RGS17+/+ mice treated with cisplatin demonstrated higher level of RGS17 immunolabeling (see red arrow) in the in OHCs, IHC and supporting Deiters cells (DCs), whereas inducible hair cell-specific RGS17 knockdown/(RGS17+/-) ameliorated cisplatin induced RGS17 immunolabeling in OHCs, IHCs and DCs. Complete inducible hair cell-specific RGS17 knockout (RGS17-/-) indicates full protection against cisplatin induced RGS17 immunolabeling in OHCs, IHCs and DCs. (B) and (C) Immunolabeling of RGS17 was increased in RGS17 wild type mice (RGS17+/+) treated with cisplatin compared to control group, while partial knockdown or complete knockout of RGS17 gene ameliorated cisplatin induced RGS17 immunolabeling in SV and SGN. Images collected from six independent animals per treatment group. Images are representative of six independent animals per treatment group. Scale bar = 20 µm. [file DataSheet1.zip › Supplementary Figure 1C.TIF]

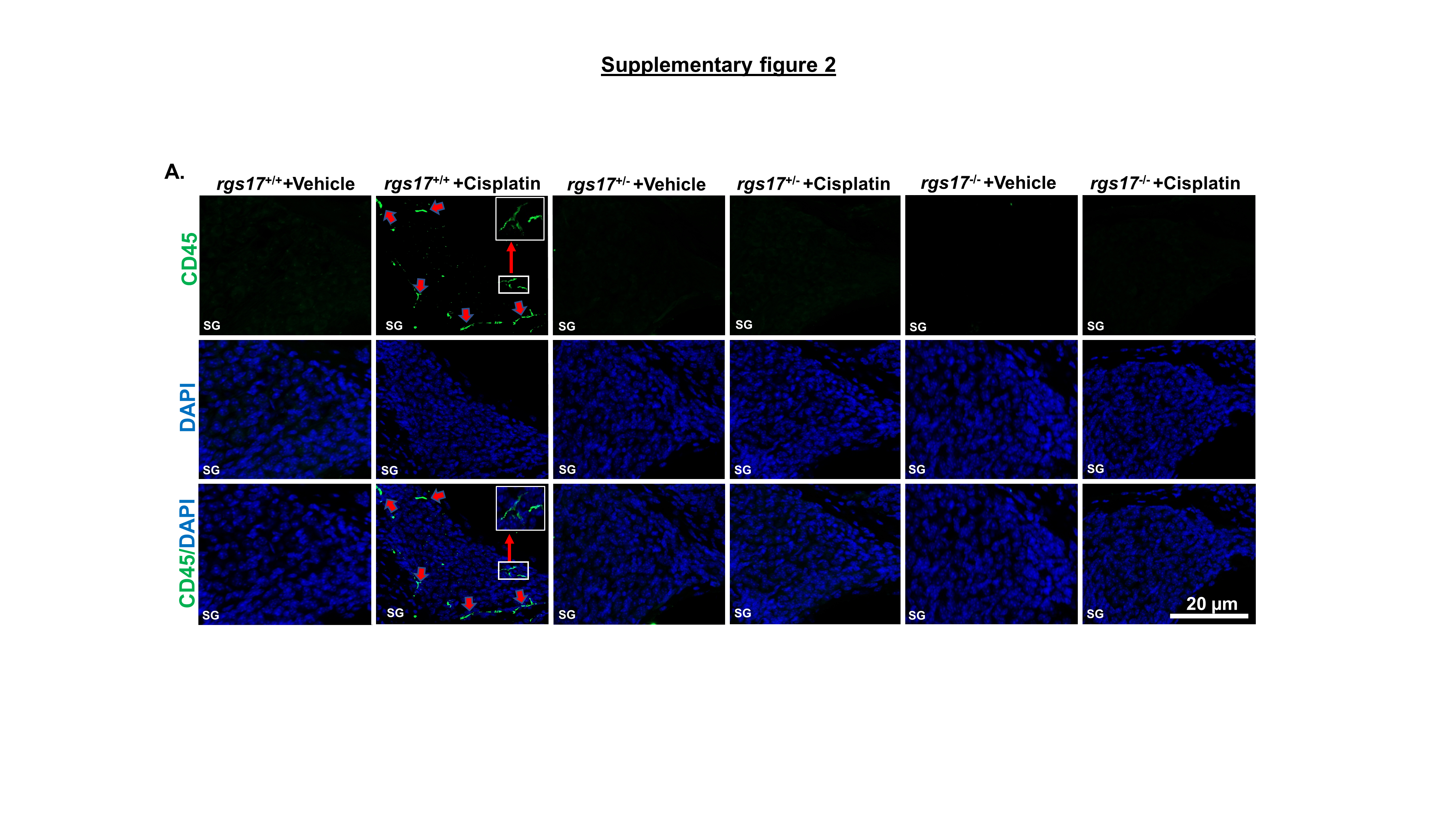

Supplement: Supplementary Figure 1 — Cisplatin administration increased RGS17 protein level in cochlear mid-modiolar sections. Mid-modiolar sections from mice treated with PBS or cisplatin (3.5 mg/kg) for two cycles were immunolabeled with RGS17 (green) and DAPI (blue). Sections were captured at high magnification to distinguish the RGS17 intensity in cochlear mid-modiolar parts. (A) RGS17+/+ mice treated with cisplatin demonstrated higher level of RGS17 immunolabeling (see red arrow) in the in OHCs, IHC and supporting Deiters cells (DCs), whereas inducible hair cell-specific RGS17 knockdown/(RGS17+/-) ameliorated cisplatin induced RGS17 immunolabeling in OHCs, IHCs and DCs. Complete inducible hair cell-specific RGS17 knockout (RGS17-/-) indicates full protection against cisplatin induced RGS17 immunolabeling in OHCs, IHCs and DCs. (B) and (C) Immunolabeling of RGS17 was increased in RGS17 wild type mice (RGS17+/+) treated with cisplatin compared to control group, while partial knockdown or complete knockout of RGS17 gene ameliorated cisplatin induced RGS17 immunolabeling in SV and SGN. Images collected from six independent animals per treatment group. Images are representative of six independent animals per treatment group. Scale bar = 20 µm. [file DataSheet1.zip › Supplementary Figure 2A.TIF]

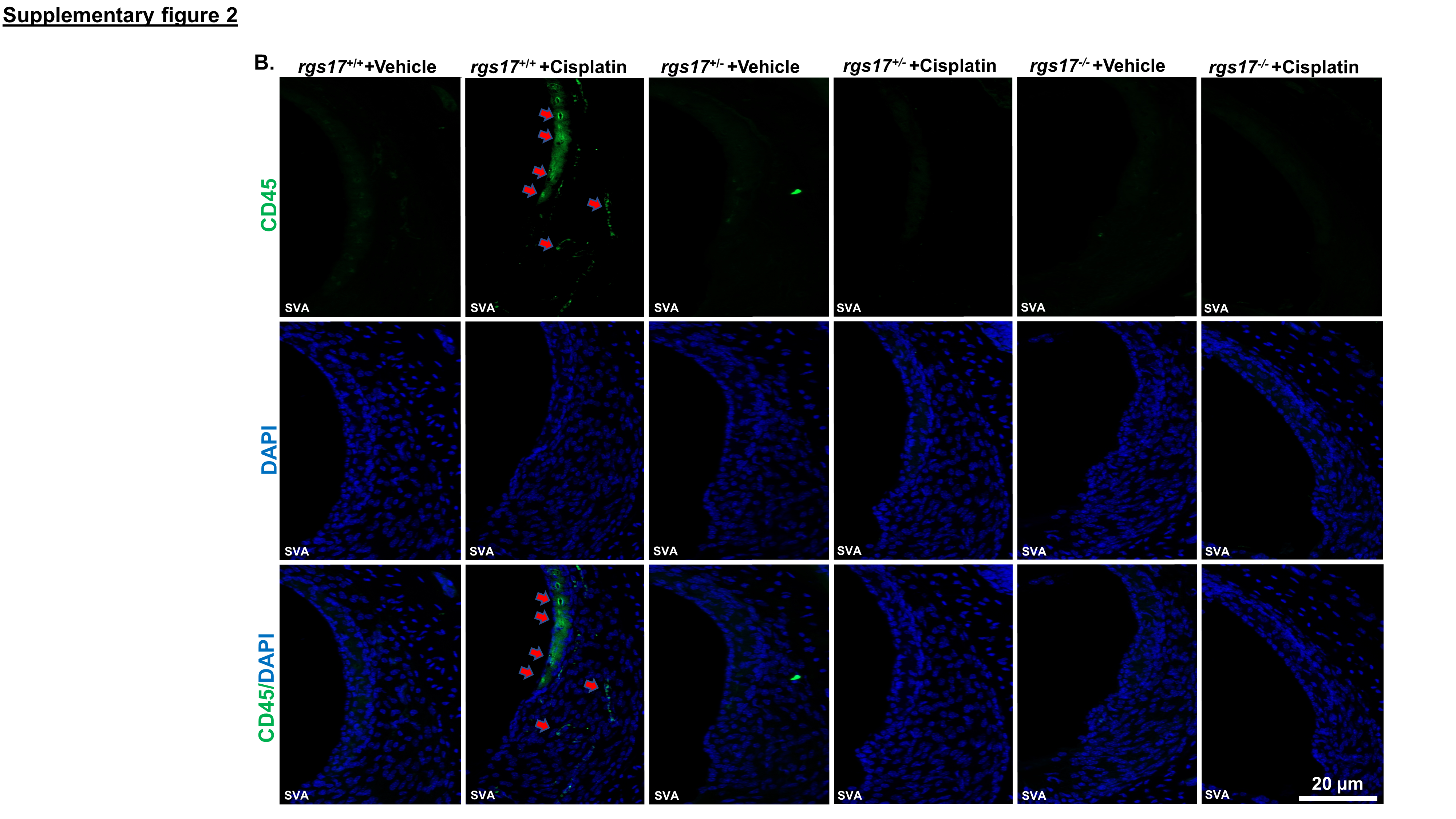

Supplement: Supplementary Figure 1 — Cisplatin administration increased RGS17 protein level in cochlear mid-modiolar sections. Mid-modiolar sections from mice treated with PBS or cisplatin (3.5 mg/kg) for two cycles were immunolabeled with RGS17 (green) and DAPI (blue). Sections were captured at high magnification to distinguish the RGS17 intensity in cochlear mid-modiolar parts. (A) RGS17+/+ mice treated with cisplatin demonstrated higher level of RGS17 immunolabeling (see red arrow) in the in OHCs, IHC and supporting Deiters cells (DCs), whereas inducible hair cell-specific RGS17 knockdown/(RGS17+/-) ameliorated cisplatin induced RGS17 immunolabeling in OHCs, IHCs and DCs. Complete inducible hair cell-specific RGS17 knockout (RGS17-/-) indicates full protection against cisplatin induced RGS17 immunolabeling in OHCs, IHCs and DCs. (B) and (C) Immunolabeling of RGS17 was increased in RGS17 wild type mice (RGS17+/+) treated with cisplatin compared to control group, while partial knockdown or complete knockout of RGS17 gene ameliorated cisplatin induced RGS17 immunolabeling in SV and SGN. Images collected from six independent animals per treatment group. Images are representative of six independent animals per treatment group. Scale bar = 20 µm. [file DataSheet1.zip › Supplementary Figure 2B.TIF]

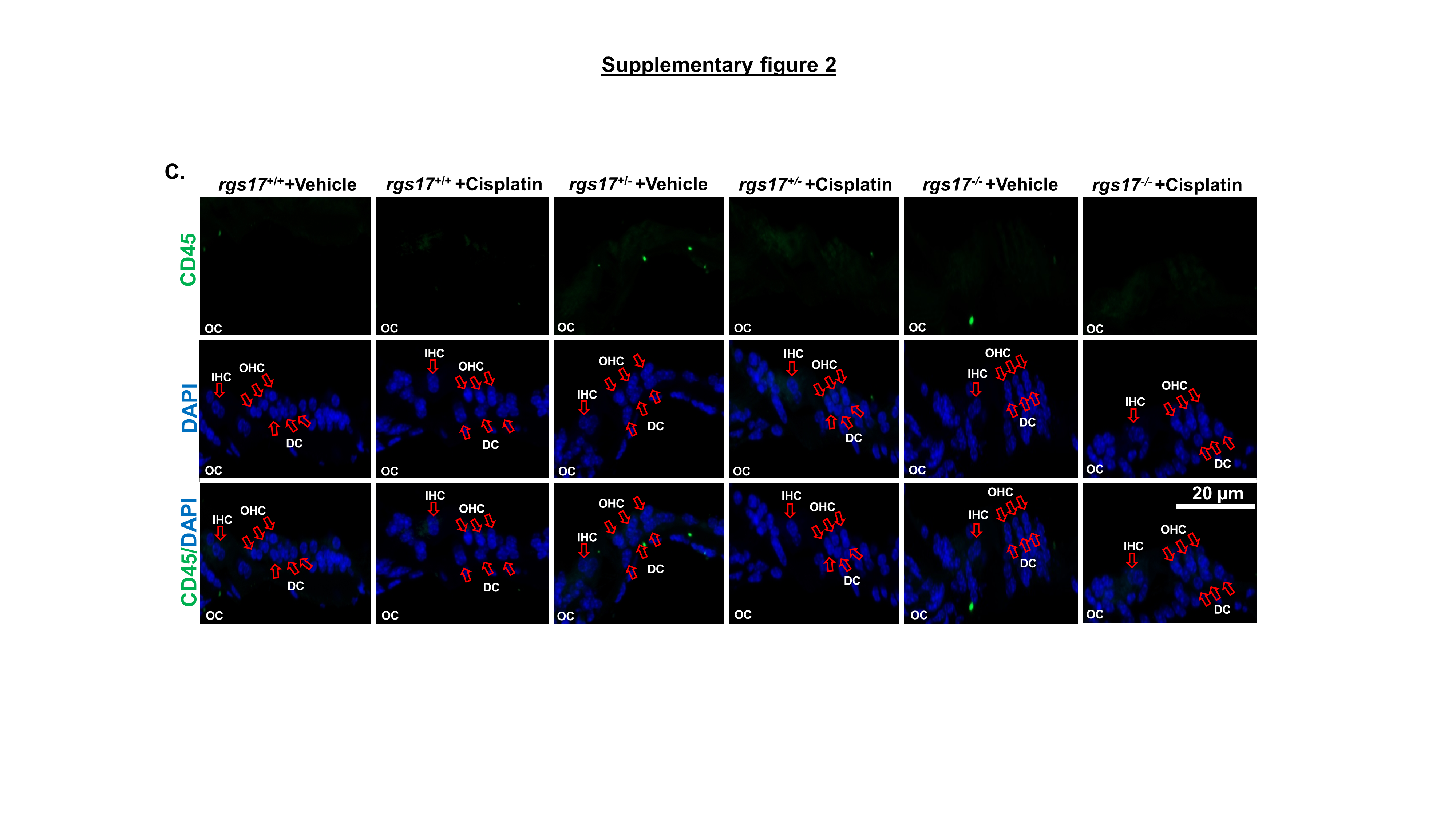

Supplement: Supplementary Figure 1 — Cisplatin administration increased RGS17 protein level in cochlear mid-modiolar sections. Mid-modiolar sections from mice treated with PBS or cisplatin (3.5 mg/kg) for two cycles were immunolabeled with RGS17 (green) and DAPI (blue). Sections were captured at high magnification to distinguish the RGS17 intensity in cochlear mid-modiolar parts. (A) RGS17+/+ mice treated with cisplatin demonstrated higher level of RGS17 immunolabeling (see red arrow) in the in OHCs, IHC and supporting Deiters cells (DCs), whereas inducible hair cell-specific RGS17 knockdown/(RGS17+/-) ameliorated cisplatin induced RGS17 immunolabeling in OHCs, IHCs and DCs. Complete inducible hair cell-specific RGS17 knockout (RGS17-/-) indicates full protection against cisplatin induced RGS17 immunolabeling in OHCs, IHCs and DCs. (B) and (C) Immunolabeling of RGS17 was increased in RGS17 wild type mice (RGS17+/+) treated with cisplatin compared to control group, while partial knockdown or complete knockout of RGS17 gene ameliorated cisplatin induced RGS17 immunolabeling in SV and SGN. Images collected from six independent animals per treatment group. Images are representative of six independent animals per treatment group. Scale bar = 20 µm. [file DataSheet1.zip › Supplementary Figure 2C.TIF]

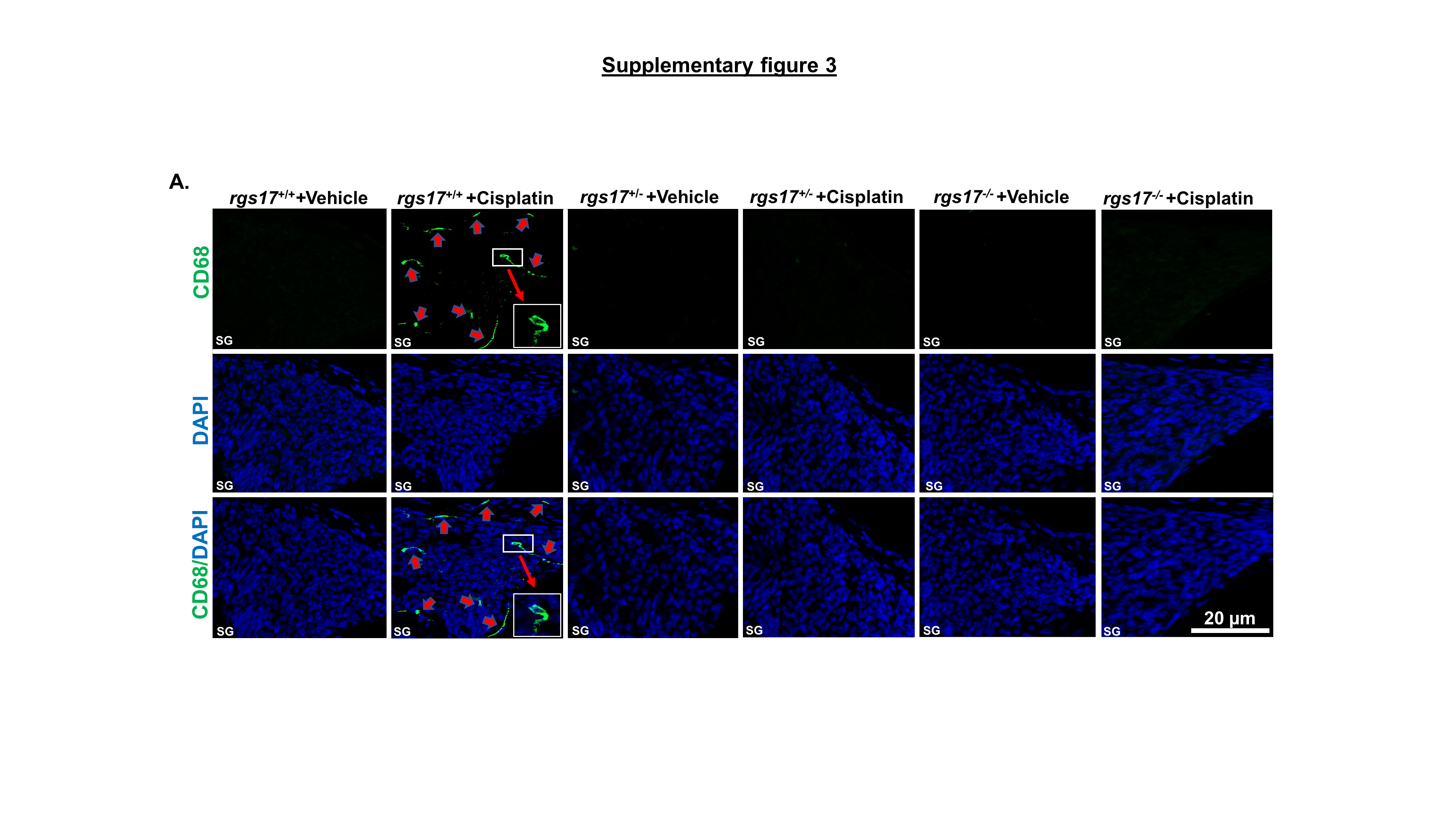

Supplement: Supplementary Figure 1 — Cisplatin administration increased RGS17 protein level in cochlear mid-modiolar sections. Mid-modiolar sections from mice treated with PBS or cisplatin (3.5 mg/kg) for two cycles were immunolabeled with RGS17 (green) and DAPI (blue). Sections were captured at high magnification to distinguish the RGS17 intensity in cochlear mid-modiolar parts. (A) RGS17+/+ mice treated with cisplatin demonstrated higher level of RGS17 immunolabeling (see red arrow) in the in OHCs, IHC and supporting Deiters cells (DCs), whereas inducible hair cell-specific RGS17 knockdown/(RGS17+/-) ameliorated cisplatin induced RGS17 immunolabeling in OHCs, IHCs and DCs. Complete inducible hair cell-specific RGS17 knockout (RGS17-/-) indicates full protection against cisplatin induced RGS17 immunolabeling in OHCs, IHCs and DCs. (B) and (C) Immunolabeling of RGS17 was increased in RGS17 wild type mice (RGS17+/+) treated with cisplatin compared to control group, while partial knockdown or complete knockout of RGS17 gene ameliorated cisplatin induced RGS17 immunolabeling in SV and SGN. Images collected from six independent animals per treatment group. Images are representative of six independent animals per treatment group. Scale bar = 20 µm. [file DataSheet1.zip › Supplementary Figure 3A.TIF]

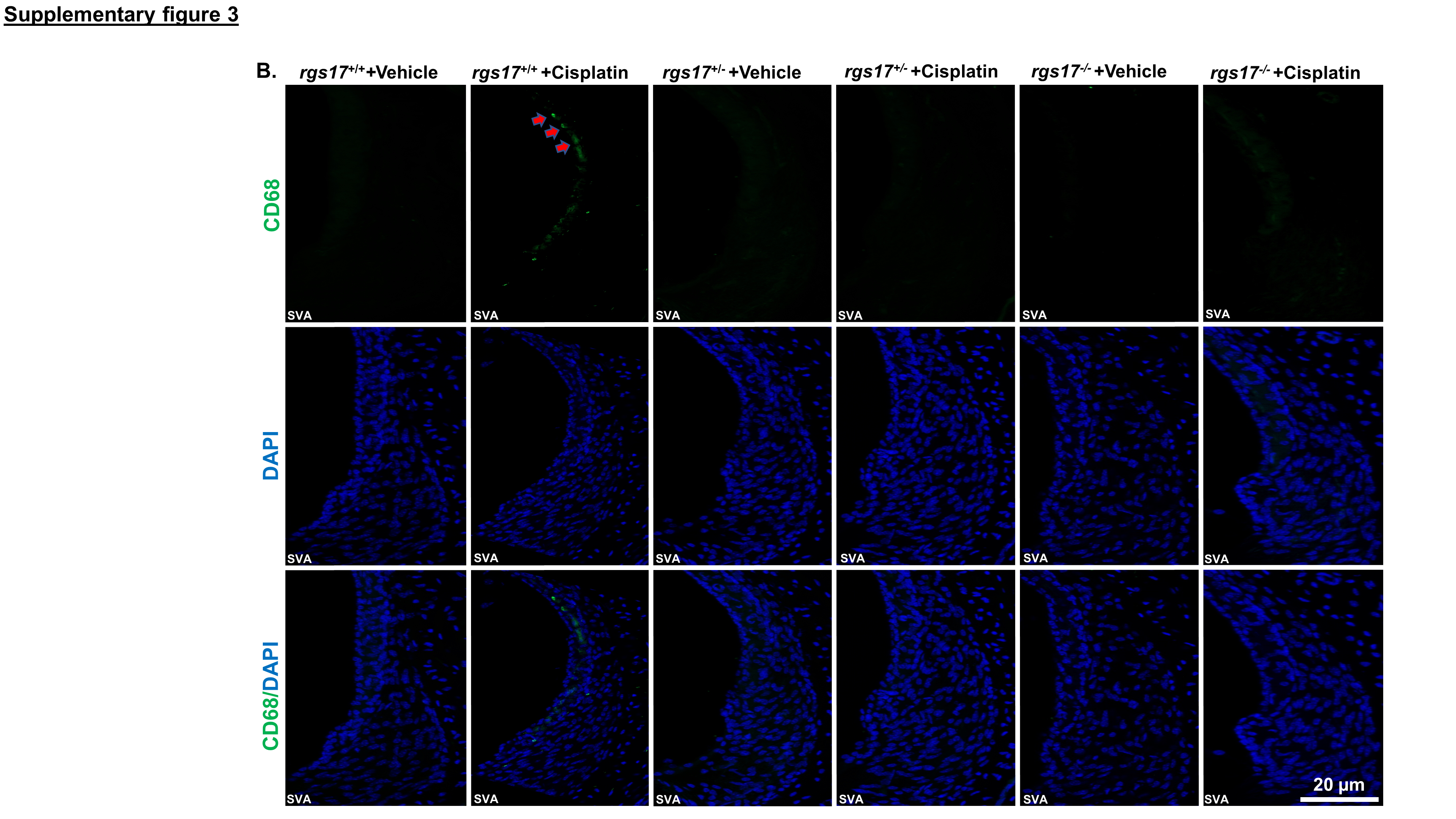

Supplement: Supplementary Figure 1 — Cisplatin administration increased RGS17 protein level in cochlear mid-modiolar sections. Mid-modiolar sections from mice treated with PBS or cisplatin (3.5 mg/kg) for two cycles were immunolabeled with RGS17 (green) and DAPI (blue). Sections were captured at high magnification to distinguish the RGS17 intensity in cochlear mid-modiolar parts. (A) RGS17+/+ mice treated with cisplatin demonstrated higher level of RGS17 immunolabeling (see red arrow) in the in OHCs, IHC and supporting Deiters cells (DCs), whereas inducible hair cell-specific RGS17 knockdown/(RGS17+/-) ameliorated cisplatin induced RGS17 immunolabeling in OHCs, IHCs and DCs. Complete inducible hair cell-specific RGS17 knockout (RGS17-/-) indicates full protection against cisplatin induced RGS17 immunolabeling in OHCs, IHCs and DCs. (B) and (C) Immunolabeling of RGS17 was increased in RGS17 wild type mice (RGS17+/+) treated with cisplatin compared to control group, while partial knockdown or complete knockout of RGS17 gene ameliorated cisplatin induced RGS17 immunolabeling in SV and SGN. Images collected from six independent animals per treatment group. Images are representative of six independent animals per treatment group. Scale bar = 20 µm. [file DataSheet1.zip › Supplementary Figure 3B.TIF]

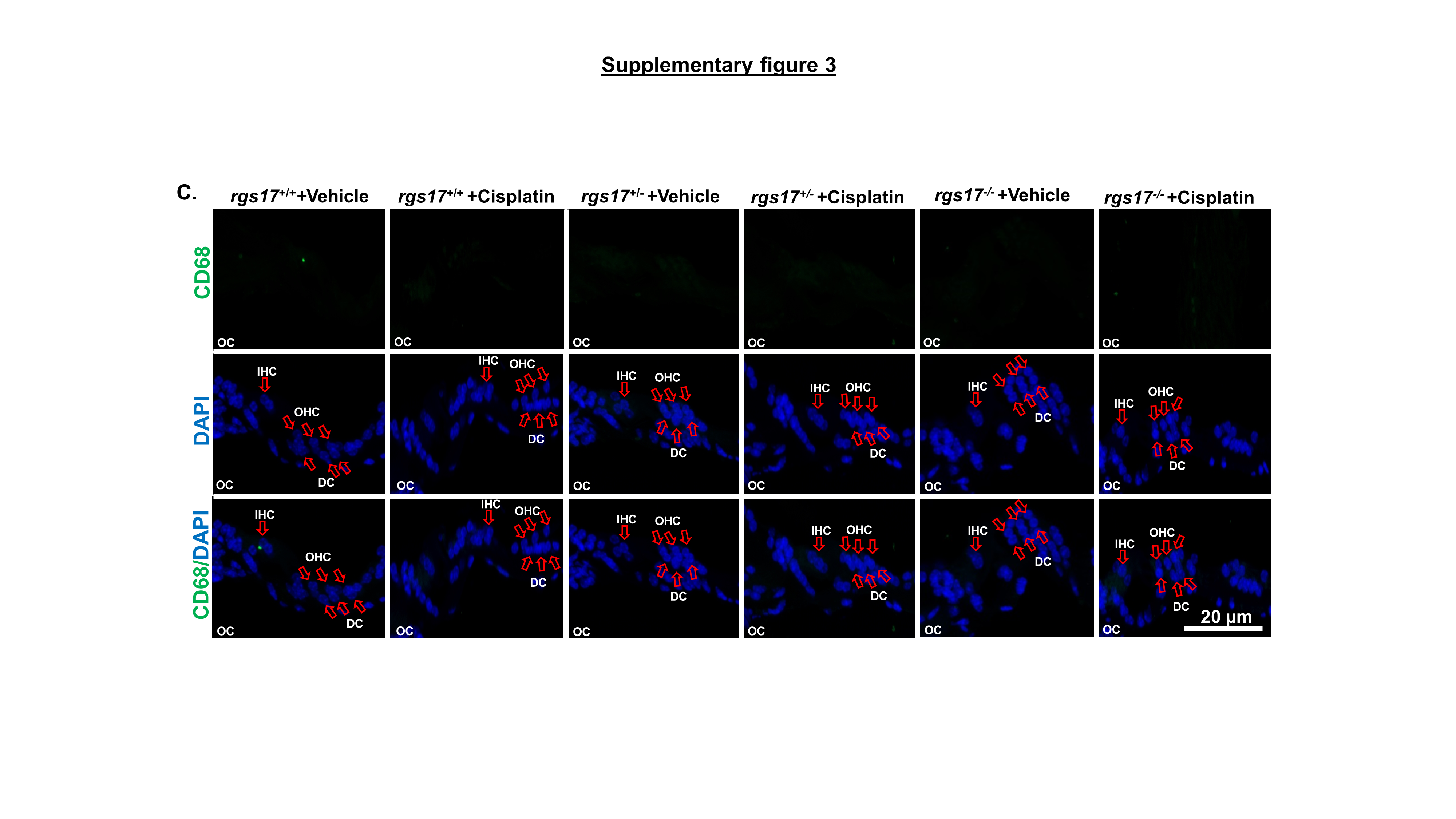

Supplement: Supplementary Figure 1 — Cisplatin administration increased RGS17 protein level in cochlear mid-modiolar sections. Mid-modiolar sections from mice treated with PBS or cisplatin (3.5 mg/kg) for two cycles were immunolabeled with RGS17 (green) and DAPI (blue). Sections were captured at high magnification to distinguish the RGS17 intensity in cochlear mid-modiolar parts. (A) RGS17+/+ mice treated with cisplatin demonstrated higher level of RGS17 immunolabeling (see red arrow) in the in OHCs, IHC and supporting Deiters cells (DCs), whereas inducible hair cell-specific RGS17 knockdown/(RGS17+/-) ameliorated cisplatin induced RGS17 immunolabeling in OHCs, IHCs and DCs. Complete inducible hair cell-specific RGS17 knockout (RGS17-/-) indicates full protection against cisplatin induced RGS17 immunolabeling in OHCs, IHCs and DCs. (B) and (C) Immunolabeling of RGS17 was increased in RGS17 wild type mice (RGS17+/+) treated with cisplatin compared to control group, while partial knockdown or complete knockout of RGS17 gene ameliorated cisplatin induced RGS17 immunolabeling in SV and SGN. Images collected from six independent animals per treatment group. Images are representative of six independent animals per treatment group. Scale bar = 20 µm. [file DataSheet1.zip › Supplementary Figure 3C.TIF]

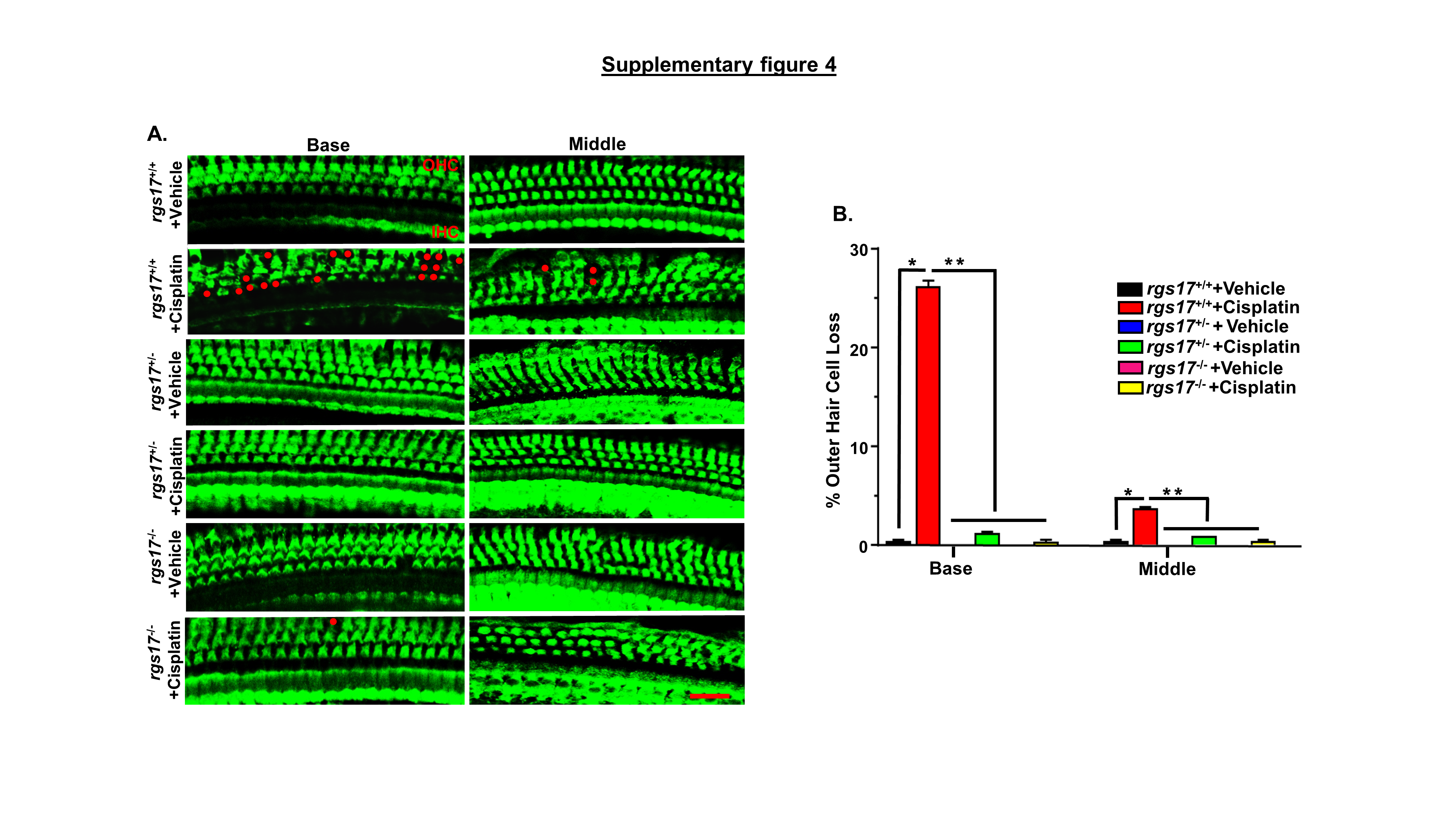

Supplement: Supplementary Figure 1 — Cisplatin administration increased RGS17 protein level in cochlear mid-modiolar sections. Mid-modiolar sections from mice treated with PBS or cisplatin (3.5 mg/kg) for two cycles were immunolabeled with RGS17 (green) and DAPI (blue). Sections were captured at high magnification to distinguish the RGS17 intensity in cochlear mid-modiolar parts. (A) RGS17+/+ mice treated with cisplatin demonstrated higher level of RGS17 immunolabeling (see red arrow) in the in OHCs, IHC and supporting Deiters cells (DCs), whereas inducible hair cell-specific RGS17 knockdown/(RGS17+/-) ameliorated cisplatin induced RGS17 immunolabeling in OHCs, IHCs and DCs. Complete inducible hair cell-specific RGS17 knockout (RGS17-/-) indicates full protection against cisplatin induced RGS17 immunolabeling in OHCs, IHCs and DCs. (B) and (C) Immunolabeling of RGS17 was increased in RGS17 wild type mice (RGS17+/+) treated with cisplatin compared to control group, while partial knockdown or complete knockout of RGS17 gene ameliorated cisplatin induced RGS17 immunolabeling in SV and SGN. Images collected from six independent animals per treatment group. Images are representative of six independent animals per treatment group. Scale bar = 20 µm. [file DataSheet1.zip › Supplementary Figure 4.TIF]

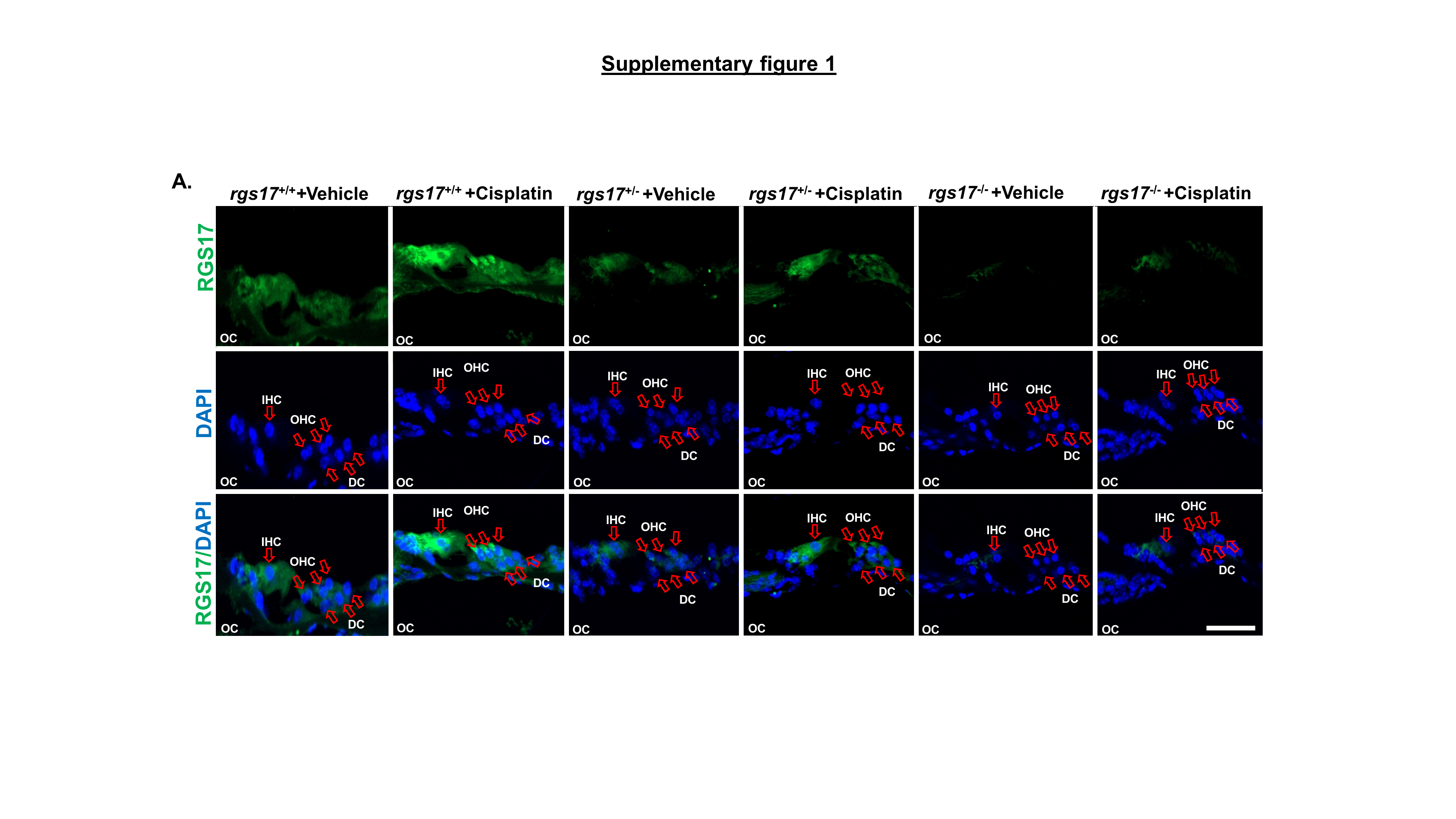

Supplement: Supplementary Figure 1 — Cisplatin administration increased RGS17 protein level in cochlear mid-modiolar sections. Mid-modiolar sections from mice treated with PBS or cisplatin (3.5 mg/kg) for two cycles were immunolabeled with RGS17 (green) and DAPI (blue). Sections were captured at high magnification to distinguish the RGS17 intensity in cochlear mid-modiolar parts. (A) RGS17+/+ mice treated with cisplatin demonstrated higher level of RGS17 immunolabeling (see red arrow) in the in OHCs, IHC and supporting Deiters cells (DCs), whereas inducible hair cell-specific RGS17 knockdown/(RGS17+/-) ameliorated cisplatin induced RGS17 immunolabeling in OHCs, IHCs and DCs. Complete inducible hair cell-specific RGS17 knockout (RGS17-/-) indicates full protection against cisplatin induced RGS17 immunolabeling in OHCs, IHCs and DCs. (B) and (C) Immunolabeling of RGS17 was increased in RGS17 wild type mice (RGS17+/+) treated with cisplatin compared to control group, while partial knockdown or complete knockout of RGS17 gene ameliorated cisplatin induced RGS17 immunolabeling in SV and SGN. Images collected from six independent animals per treatment group. Images are representative of six independent animals per treatment group. Scale bar = 20 µm. [file DataSheet1.zip › Supplementary Figure 1A.TIF]

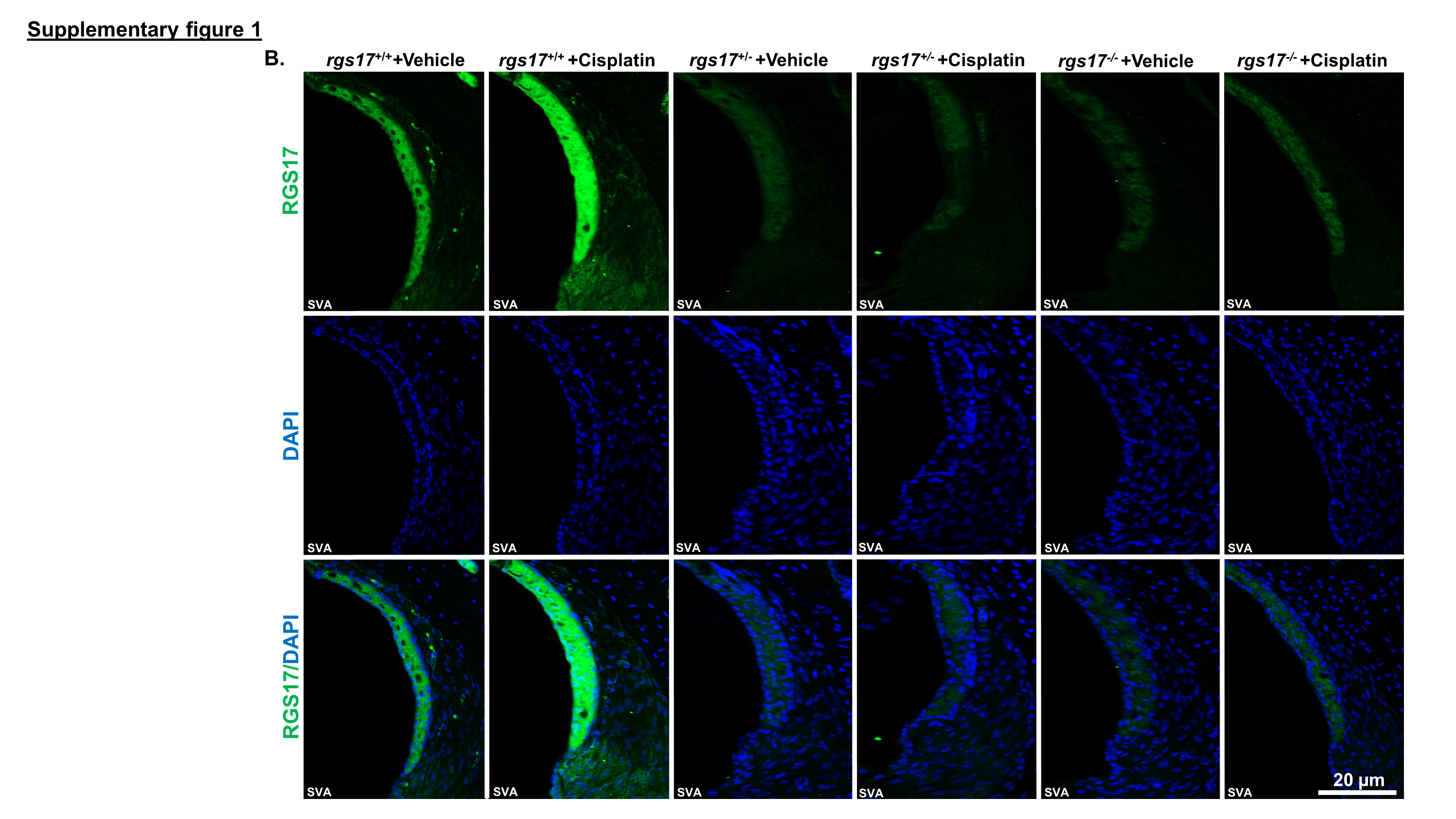

Supplement: Supplementary Figure 1 — Cisplatin administration increased RGS17 protein level in cochlear mid-modiolar sections. Mid-modiolar sections from mice treated with PBS or cisplatin (3.5 mg/kg) for two cycles were immunolabeled with RGS17 (green) and DAPI (blue). Sections were captured at high magnification to distinguish the RGS17 intensity in cochlear mid-modiolar parts. (A) RGS17+/+ mice treated with cisplatin demonstrated higher level of RGS17 immunolabeling (see red arrow) in the in OHCs, IHC and supporting Deiters cells (DCs), whereas inducible hair cell-specific RGS17 knockdown/(RGS17+/-) ameliorated cisplatin induced RGS17 immunolabeling in OHCs, IHCs and DCs. Complete inducible hair cell-specific RGS17 knockout (RGS17-/-) indicates full protection against cisplatin induced RGS17 immunolabeling in OHCs, IHCs and DCs. (B) and (C) Immunolabeling of RGS17 was increased in RGS17 wild type mice (RGS17+/+) treated with cisplatin compared to control group, while partial knockdown or complete knockout of RGS17 gene ameliorated cisplatin induced RGS17 immunolabeling in SV and SGN. Images collected from six independent animals per treatment group. Images are representative of six independent animals per treatment group. Scale bar = 20 µm. [file DataSheet1.zip › Supplementary Figure 1B.TIF]
